# Synthesis and Radioprotective Activity of Benzyl Sulfoxide/Sulfone Coumarins Derived from Ex-RAD

**DOI:** 10.3390/molecules31030487

**Published:** 2026-01-30

**Authors:** Tao Wang, Chunrui Zhou, Ningfan Liu, Tao Peng, Lin Wang, Shouguo Zhang

**Affiliations:** 1 School of Chemistry and Chemical Engineering, Henan Institute of Science and Technology, Xinxiang 453003, China; zhouchunruihist@163.com (C.Z.); 17630204160@163.com (N.L.); 2 Beijing Institute of Radiation Medicine, Beijing 100850, China; peng-tao@sohu.com

**Keywords:** radioprotection, coumarin derivatives, alkaline comet assay, mice survival

## Abstract

In order to identify promising radioprotector candidates, a series of benzyl sulfoxide/sulfone coumarin derivatives were designed and synthesized based on the reported activity of Ex-Rad. Most of the target compounds demonstrated significant radioprotective effects at concentrations of 40 μmol/L and 20 μmol/L in HUVECs. Among them, compounds **5q** and **5u** displayed superior activity in mitigating DNA damage compared with Ex-RAD. Notably, compound **5u** significantly enhanced the viability of mice exposed to a lethal dose of ionizing radiation. In summary, the above findings suggested that compound **5u** might be a promising radioprotective agent deserving further investigation.

## 1. Introduction

Currently, ionizing radiation (IR) is widely used in modern medicine for purposes such as radio-diagnosis and radiotherapy of cancer [1,2]. However, long-term or high-level exposure to IR can lead to adverse effects, including DNA damage, cell death, and even multi-organ dysfunction [3,4]. Therefore, protecting biological systems from radiation-induced damage is of considerable importance [5,6].

In the category of chemical pharmaceuticals, particularly sulfur-containing compounds, amifostine (WR2721) remains the only radioprotective agent approved by the U.S. FDA for clinical use against radiotherapy-induced side effects [7,8]. However, its application is limited by considerable adverse effects, including vomiting, nausea, and hypotension [9,10]. Therefore, there is a clear need to develop novel radioprotectors that are less toxic, more effective, and suitable for self-administration.

Ex-RAD (ON 01210.Na), characterized chemically as the sodium salt of 4-carboxystyryl-4-chlorobenzylsulfone, is a promising radioprotective agent developed by Onconova Therapeutics (Newtown, PA, USA) [11]. Unlike most conventional radioprotectors, Ex-RAD does not act primarily as a free-radical scavenger; instead, it exhibits a novel mechanism involving drug-mediated enhancement of DNA repair and cell survival pathways [12]. In vitro studies have shown that Ex-RAD provides strong radioprotection in several human cell lines [13]. In vivo, Ex-RAD has been effective in improving survival rates at cellular, tissue, and organ levels across various animal models of radiation injury, consistent with its proposed mechanism [11,14]. Onconova Therapeutics has assessed Ex-RAD in four Phase I trials involving more than 60 healthy volunteers, with no reported systemic side effects [15].

**Figure 1:**
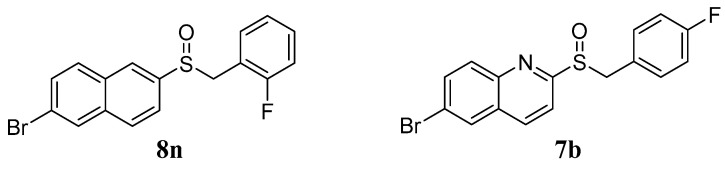
The structures of compounds **8n** and **7b**.

In our previous studies, a series of benzyl naphthyl sulfoxide (sulfone) and benzyl quinoline sulfoxide (sulfone) derivatives were designed based on the structural modification of Ex-RAD [16,17]. Among these derivatives, compounds **8n** and **7b** (Figure 1) demonstrated notably radioprotective efficacy compared with Ex-RAD, which was reflected in the enhancement of cell survival, reduction in DNA damage, and improved survival in mice following exposure to ^60^Co γ-irradiation.

**Figure 2:**
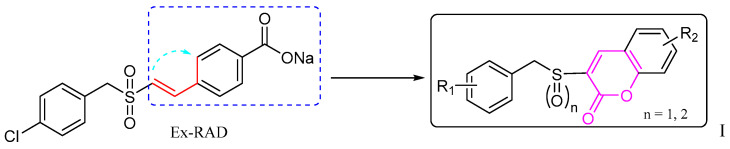
The design of the new benzyl sulfoxide/sulfone coumarins (I). The dashed blue box, the red line with a blue arrow, and the black box indicate the modified benzyl vinyl segment, the specific site of modification and connection, and the resulting compound, respectively.

Based on this background, it seemed worthwhile to design and synthesize certain new derivatives by backbone modification of Ex-RAD, with the aim of obtaining compounds exhibiting enhanced radioprotective activity. In this study, bioisosterism was used to modify the structure of styrylbenzylsulfones. A series of benzyl sulfoxide/sulfone coumarins were obtained by rationally linking the *ortho*-position of styrene benzene ring to the 2-position of vinyl rationally (Figure 2).

In recent years, coumarins and their derivatives have attracted considerable interest owing to their diverse biological activities, including anticancer [18], antibacterial [19], antioxidant [20] and anti-radiation effects [21].

Herein, this study reports the design, synthesis, and radioprotective evaluation of novel coumarin derivatives derived from Ex-RAD, aming to discover promising candidates with potent efficacy.

## 2. Results and Discussion

### 2.1. Chemistry

**Scheme 1:**
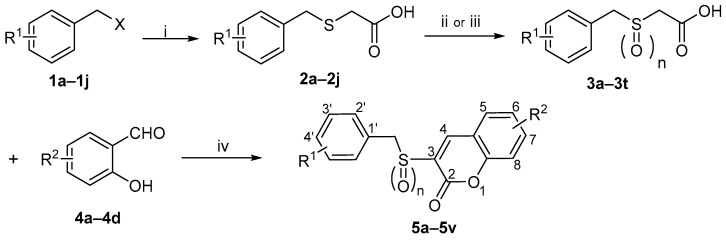
Reagents and Conditions: (i) HSCH_2_COOH, NaOH, CH_3_OH, rt, 1–2 h, 58–90%; (ii) H_2_O_2_, NaOH, H_2_O, rt, 2 h, 65–89%; (iii) H_2_O_2_, CH_3_COOH, 55 °C, 4 h, 70–93%; (iv) EDCI, DMAP, CH_3_CN, rt, 1 h, 35–57%.

The synthesis of target compounds **5a**–**5v** followed the three-step route shown in Scheme 1, commencing with substituted benzyl chlorides/bromides (**1a**–**1j**). The procedure involved: (i) reaction of **1a**–**1j** with mercaptoacetic acid under basic conditions to afford acids **2a**–**2j** [22]; (ii) oxidation of **2a**–**2j** with 30% H_2_O_2_ to give sulfonylacetic acids **3a**–**3t** [23]; and (iii) in the final step, a Knoevenagel condensation [24,25] between **3a**–**3t** and salicylaldehydes **4a**–**4d**, promoted by 1-(3-Dimethylaminopropyl)-3- ethylcarbodiimide hydrochloride (EDCI) and 4-dimethylaminopyridine (DMAP), to furnish **5a**–**5v**. Following purification via recrystallization, the structures of all final compounds were unequivocally confirmed by ^1^H-NMR, ^13^C-NMR and HRMS spectra analyses.

**1a**: R^1^ = H, X = Cl**2g**: R^1^ = 2-Br**3m**: R^1^ = 3-Cl, *n* = 2**5f**: R^1^ = 4′-CH_3_, R^2^ = H, *n* = 2**1b**: R^1^ = 2-Cl, X = Br**2h**: R^1^ = 4-Br**3n**: R^1^ = 2-F, *n* = 2**5g**: R^1^ = 2’-F, R^2^ = 6-NO_2_, *n* = 1**1c**: R^1^ = 3-Cl, X = Br**2i**: R^1^ = 3-OCH_3_**3o**: R^1^ = 3-F, *n* = 2**5h:** R^1^ = 2′-F, R^2^ = 6-NO_2_, *n* = 2**1d**: R^1^ = 2-F, X = Cl**2j**: R^1^ = 4-CH_3_**3p**: R^1^ = 4-F, *n* = 2**5i:** R^1^ = 3′-F, R^2^ = 6-NO_2_, *n* = 1**1e**: R^1^ = 3-F, X = Br**3a**: R^1^ = H, *n* = 1**3q:** R^1^ = 2-Br, *n* = 2**5j:** R^1^ = 3′-F, R^2^ = 6-NO_2_, *n* = 2**1f**: R^1^ = 4-F, X = Br**3b**: R^1^ = 2-Cl, *n* = 1**3r:** R^1^ = 4-Br, *n* = 2**5k:** R^1^ = 4′-F, R^2^ = 6-NO_2_, *n* = 1**1g**: R^1^ = 2-Br, X = Cl**3c**: R^1^ = 3-Cl, *n* = 1**3s:** R^1^ = 3-OCH_3_, *n* = 2**5l:** R^1^ = 4′-F, R^2^ = 6-NO_2_, *n* = 2**1h**: R^1^ = 4-Br, X = Br**3d**: R^1^ = 2-F, *n* = 1**3t:** R^1^ = 4-CH_3_, *n* = 2**5m**: R^1^ = 4′-Br, R^2^ = 6-NO_2_, *n* = 1**1i**: R^1^ = 3-OCH_3_, X = Br**3e**: R^1^ = 3-F, *n* = 1**4a**: R^2^ = H**5n**: R^1^ = 4′-Br, R^2^ = 6-NO_2_, *n* = 2**1j**: R^1^ = 4-CH_3_, X = Cl**3f**: R^1^ = 4-F, *n* = 1**4b**: R^2^ = 5-NO_2_**5o**: R^1^ = 3′-OCH_3_, R^2^ = 6-NO_2_, *n* = 1**2a**: R^1^ = H**3g**: R^1^ = 2-Br, *n* = 1**4c**: R^2^ = 3-OCH_3_**5p**: R^1^ = 3′-OCH_3_, R^2^ = 6-NO_2_, *n* = 2**2b**: R^1^ = 2-Cl**3h:** R^1^ = 4-Br, *n* = 1**4d**: R^2^ = 4-OCH_3_**5q**: R^1^ = 4′-CH_3_, R^2^ = 6-NO_2_, *n* = 1**2c**: R^1^ = 3-Cl**3i:** R^1^ = 3-OCH_3_, *n* = 1**5a**: R^1^ = H, R^2^ = H, *n* = 1**5r**: R^1^ = 4′-CH_3_, R^2^ = 6-NO_2_, *n* = 2**2d**: R^1^ = 2-F**3j:** R^1^ = 4-CH_3_, *n* = 1**5b**: R^1^ = H, R^2^ = H, *n* = 2**5s:** R^1^ = 2′-Cl, R^2^ = 7-OCH_3_, *n* = 2**2e**: R^1^ = 3-F**3k**: R^1^ = H, *n* = 2**5c**: R^1^ = 3′-Cl, R^2^ = H, *n* = 1**5t:** R^1^ = 2′-Cl, R^2^ = 8-OCH_3_, *n* = 2**2f**: R^1^ = 4-F**3l**: R^1^ = 2-Cl, *n* = 2**5d**: R^1^ = 3′-Cl, R^2^ = H, *n* = 2**5u:** R^1^ = 4′-Br, R^2^ = 8-OCH_3_, *n* = 2

**5e**: R^1^ = 4′-CH_3_, R^2^ = H, *n* = 1**5v:** R^1^ = 2′-Br, R^2^ = 8-OCH_3_, *n* = 2

### 2.2. Antiradiation Activity Evaluation

#### 2.2.1. In Vitro Evaluation

None of the target compounds showed evident toxicity at a concentration of 100 μmol/L in HUVECs. In line with our prior biological activity findings [16], the radioprotective effects were primarily evaluated in vitro using an irradiation assay in HUVECs. Cells were pretreated with the target compounds 24 h prior to exposure to ^60^Co γ-irradiation (8.0 Gy), with Ex-RAD serving as a positive control. Post-irradiation cell viability was evaluated via the MTS tetrazolium assay [26].

**Figure 3:**
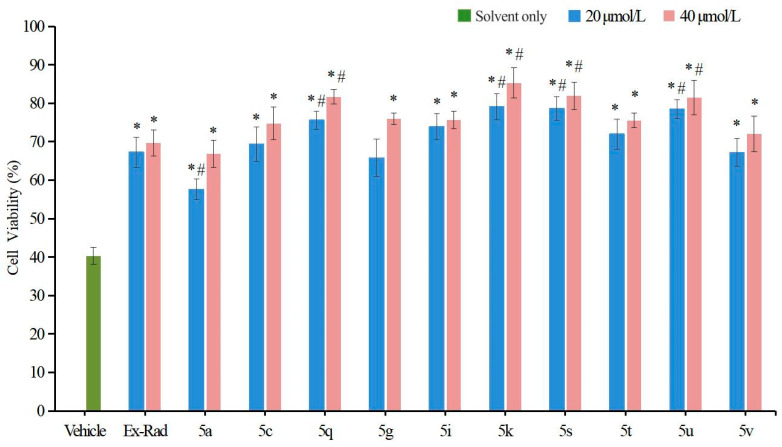
Cell viability in compound-pretreated cells after 24 h irradiation. Radiation dosage, 8 Gy. The green bar indicates the vehicle control (solvent only). Compounds dosage was 40 μmol/L (red bar) and 20 μmol/L (blue bar), respectively. * *p* < 0.05, vs. the vehicle; ^#^ *p* < 0.05, vs. the Ex-RAD. Data presented as mean ± standard deviation.

Among the 22 target compounds, half exhibited pronounced radioprotective activity at concentrations of 40 μmol/L and 20 μmol/L compared with the vehicle control (*p* < 0.05, Figure 3). Notably, four compounds (**5k**, **5q**, **5s**, **5u**) significantly improved cell viability relative to Ex-RAD (*p* < 0.05).

These in vitro results indicate that several target compounds afford superior radioprotection compared with Ex-RAD at various concentrations, thereby supporting the coumarin scaffold as a key structural motif and confirming the feasibility of our design strategy. When comparing activities based on the sulfur oxidation state, sulfoxide derivatives generally exhibited higher bioactivity than their corresponding sulfone analogs. For instance, at a concentration of 20 μmol/L, sulfoxide-containing compounds **5c** (69.36% viability) and **5i** (73.96% viability) showed more pronounced radioprotective effects than sulfones **5d** (43.52%) and **5j** (45.29%). This may be attributed to the presence of sulfoxide and the substituents on the coumarin ring, which could together enhance steric complementarity with the target protein.

In summary, compounds **5q** (75.58%, 81.71% viability), **5k** (79.13%, 85.30% viability), **5s** (78.67%, 81.89% viability) and **5u** (78.55%, 81.48% viability) displayed statistically significant differences (*p* < 0.05) compared with Ex-RAD (65.29%, 69.68% viability) at concentrations of 20 μmol/L and 40 μmol/L. However, the underlying mechanism remains unclear, warranting further investigation of these promising candidates.

#### 2.2.2. Protective Effects of Target Compounds on Radiation-Induced DNA Damage

Exposure to ionizing radiation leads to DNA damage, which can be detected as an increase in comet tail intensity using the alkaline comet assay—a well-established method for quantifying DNA damage at the single-cell level [27]. In this study, an in vitro alkaline comet assay was performed to assess the protective capacity of various compounds against radiation-induced DNA fragmentation. HUVECs were irradiated with ^60^Co γ-rays (6.0 Gy). Following staining with Vista Green DNA dye, seventy randomly chosen comets were imaged by fluorescence microscopy. DNA damage was quantified by calculating tail lengths, percentage of DNA in tail and Olive Tail Moment (OTM) using the Comet Assay Software Project Lab (version 1.2.3).

**Figure 4:**
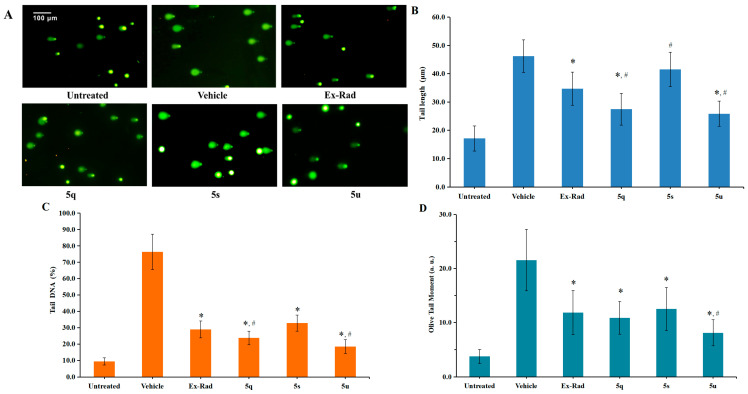
DNA damage in HUVECs was assessed using the alkaline comet assay. Normally cultured cells without irradiation served as the untreated control group. (**A**). Representative fluorescence microscopy images of HUVECs taken 24 h after exposure to 6.0 Gy irradiation. (**B**). The extent of DNA damage was quantified based on tail lengths measured at 24 h post-irradiation. (**C**). DNA damage is also expressed as percentage of DNA in tail. (**D**). Quantitative analysis of DNA damage expressed as Olive Tail Moment (a. u.). * *p* < 0.05, vs. the vehicle; ^#^ *p* < 0.05, vs. the Ex-RAD. Data presented as mean ± standard deviation.

As shown in Figure 4, irradiation caused a marked elevation in tail length, Tail DNA (%) and OTM in control cells. In contrast, pretreatment with the target compounds **5q** (27.25 μm, 10.88 OTM) and **5u** (25.88 μm, 8.10 OTM) led to significantly shorter tail lengths and lower OTM values in HUVECs compared to the vehicle control group (46.17 μm, 21.55 OTM; *p* < 0.05). Furthermore, the protective effect against DNA damage was more pronounced for **5q** (Tail DNA: 23.77%) and **5u** (Tail DNA: 18.44%) than for the reference compound Ex-RAD (Tail DNA: 28.99%; *p* < 0.05). These findings demonstrate that compounds **5q** and **5u** play a critical role in repairing DNA damage following ionizing radiation exposure.

#### 2.2.3. Radioprotective Efficacy of **5q** and **5u** In Vivo

**Figure 5:**
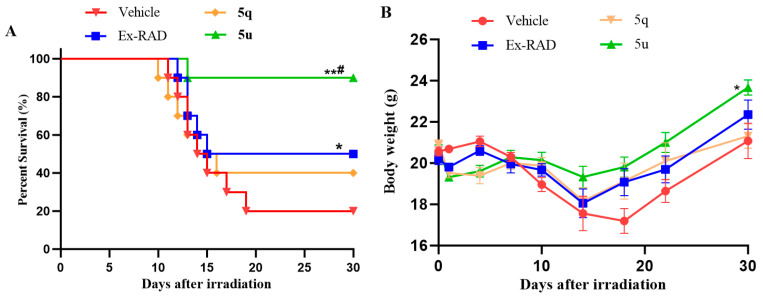
(**A**) The 30-day survival was assessed by the Kaplan–Meier method in male C57BL/6 mice (n = 10 per group). Administration of **5u** resulted in significantly higher survival (90%) compared with both the vehicle control and Ex-RAD groups. * *p* < 0.05, ** *p* < 0.01 vs. the control group; # *p* < 0.05 vs. the Ex-RAD. (**B**) Body weights were measured in male C57BL/6 mice (10 per group) at 30 days post-irradiation. From day 14 to day 30 after irradiation, mice treated with **5u** showed a statistically significant increase in body weight (*p* < 0.05) relative to the control and Ex-RAD groups.

In vitro studies demonstrated significant radioprotective effects of compounds **5q** and **5u**. These effects were further evaluated in vivo using C57BL/6 male mice [28]. The 30-day survival following 8.0 Gy ^60^Co γ-irradiation was evaluated, with Ex-RAD serving as a positive control. As shown in Figure 5A, administration of compound **5u** led to a substantially higher survival rate (90%) compared to the vehicle (20%) and Ex-RAD (50%) groups. In contrast, the survival rate for the **5q**-treated group was 40%. Additionally, **5u** treatment was associated with higher body weights from days 14 to 30 post-irradiation relative to the vehicle and Ex-RAD groups (Figure 5B).

## 3. Materials and Methods

### 3.1. Materials and General Methods

All the reagents and solvents were purchased from commercial sources (Beijing Innochem Technology Co., Ltd., Beijing, China) and used without further purification, unless otherwise stated. Ex-RAD was synthesized and characterized by our research group following a literature procedure [13]. Reaction progress was tracked using thin-layer chromatography (TLC) performed on pre-coated silica gel F254 plates. Melting points were determined with a Uniscience Melting Point apparatus and were uncorrected. ^1^H-NMR and ^13^C-NMR spectra were obtained on Bruker AM 400 and AM 500 MHz spectrometers (Palo Alto, CA, USA). High-resolution mass spectra (HRMS) were recorded via electrospray ionization on a Micromass ZabSpec spectrometer (Karlsruhe, Germany). Chemical shifts (δ) are reported in parts per million relative to tetramethylsilane (Me_4_Si) as an internal standard. Spin multiplicities are indicated as s (singlet), d (doublet), m (multiplet), and q (quartet). Coupling constants (*J*) are provided in hertz (Hz).

Note: This article focuses on the synthesis and characterization of the target compounds. Detailed data for the intermediates outlined in Scheme 1 are included in the Supplementary Materials.

### 3.2. Synthesis of the Target Compounds *(**5a***–***5v**)*

To an ice-cooled solution of the substituted benzyl sulfonyl/sulfoxy acetic acid derivatives (**3a**–**3t**, 1.0 equiv.) in acetonitrile, substituted salicylaldehydes (**4a**–**5d**, 1.05 equiv.), EDCI (2.1 equiv.), and DMAP (0.1 equiv.) were added [29]. The mixture was stirred at room temperature for 1 h, at which point TLC analysis indicated complete consumption of the starting materials. The precipitated solid was collected by filtration, redissolved in CH_2_Cl_2_, and washed successively with saturated Na_2_CO_3_ solution, dilute hydrochloric acid, and saturated brine. The organic layer was then dried over anhydrous Na_2_SO_4_. After concentration under reduced pressure, the resulting crude material was recrystallized from ethyl acetate to afford the target **5a**–**5v**.

It should be noted that compounds **5h**, **5j**, **5l** are known compounds, and their syntheses have been reported previously by our team [29,30].

3-(Benzylsulfinyl)-2H-chromen-2-one (**5a**)

Yield 45%, white solid, m.p. 159–161 °C. ^1^H-NMR (400 MHz, DMSO-*d*_6_) δ: 4.11 (d, 1H, *J* = 13.2 Hz, -CH_2_-), 4.54 (d, 1H, *J* = 13.2 Hz, -CH_2_-), 7.13–7.16 (m, 2H, Ar-H), 7.28 (m, 3H, Ar-H), 7.40 (t, 1H, *J* = 7.6 Hz, Ar-H), 7.53 (d, 1H, *J* = 8.4 Hz, Ar-H), 7.70 (t, 1H, *J* = 7.6 Hz, Ar-H), 7.85 (d, 1H, *J* = 7.6 Hz, Ar-H), 8.03 (s, 1H, =CH-). ^13^C-NMR (101 MHz, DMSO-*d*_6_) δ: 57.6, 116.9, 118.7, 125.7, 128.6, 128.7, 129.8, 130.2, 130.9, 131.5, 134.0, 143.1, 153.9, 157.5. HRMS-ESI (*m*/*z*): calcd for C_16_H_12_O_3_S [M + H]^+^ 285.0585, found: 285.0579.

3-(Benzylsulfonyl)-2H-chromen-2-one (**5b**)

Yield 42%, white solid, m.p. 169–172 °C. ^1^H-NMR (400 MHz, DMSO-d_6_) δ: 4.83 (s, 2H, -CH_2_-), 7.31–7.39 (m, 5H, Ar-H), 7.46 (t, 1H, *J* = 7.6 Hz, Ar-H), 7.56 (d, 1H, *J* = 8.0 Hz, Ar-H), 7.83 (t, 1H, *J* = 8.0 Hz, Ar-H), 8.00 (d, 1H, *J* = 8.0 Hz, Ar-H), 8.76 (s, 1H, =CH-). ^13^C-NMR (101 MHz, DMSO-*d*_6_) δ: 58.0, 117.0, 118.9, 125.9, 128.7, 129.2, 129.8, 130.4, 130.7, 131.2, 133.9, 144.2, 153.2, 159.6. HRMS-ESI (*m*/*z*): calcd for C_16_H_12_O_4_S [M + H]^+^ 301.0535, found: 301.0529.

3-((3-Chlorobenzyl)sulfinyl)-2H-chromen-2-one (**5c**)

Yield 39%, white solid, m.p. 174–176 °C. ^1^H-NMR (400 MHz, DMSO-*d*_6_) δ: 4.16 (d, 1H, *J* = 13.2 Hz, -CH_2_-), 4.58 (d, 1H, *J* = 13.2 Hz, -CH_2_-), 7.07 (d, 1H, *J* = 6.0 Hz, Ar-H), 7.21 (s, 1H, Ar-H), 7.30 (t, 1H, *J* = 8.0 Hz, Ar-H), 7.34 (t, 1H, *J* = 8.0 Hz, Ar-H), 7.41 (t, 1H, *J* = 8.0 Hz, Ar-H), 7.54 (d, 1H, *J* = 8.4 Hz, Ar-H), 7.72 (t, 1H, *J* = 8.0 Hz, Ar-H), 7.88 (d, 1H, *J* = 8.0 Hz), 8.03 (s, 1H, =CH-). ^13^C-NMR (101 MHz, DMSO-*d*_6_) δ: 56.0, 116.4, 118.2, 125.2, 128.0, 129.1, 129.4, 130.0, 130.3, 130.6, 131.9, 132.7, 133.6, 142.8, 153.4, 156.9. HRMS-ESI (*m*/*z*): calcd for C_16_H_11_ClO_3_S [M + H]^+^ 319.0196, found: 319.0190.

3-((3-Chlorobenzyl)sulfonyl)-2H-chromen-2-one (**5d**)

Yield 43%, white solid, m.p. 199–201 °C. ^1^H-NMR (400 MHz, DMSO-d_6_) δ: 4.87 (s, 2H, -CH_2_-), 7.29 (d, 1H, *J* = 7.6 Hz, Ar-H), 7.39 (t, 1H, *J* = 8.0 Hz, Ar-H), 7.42–7.49 (m, 3H, Ar-H), 7.57 (d, 1H, *J* = 8.0 Hz, Ar-H), 7.85 (t, 1H, *J* = 8.0 Hz, Ar-H), 8.03 (d, 1H, *J* = 8.0 Hz, Ar-H), 8.81 (s, 1H, =CH-). ^13^C-NMR (101 MHz, DMSO-*d*_6_) δ: 56.5, 116.3, 118.4, 125.7, 128.5, 129.0, 129.5, 130.3, 130.5, 130.9, 132.6, 133.4, 142.5, 153.6, 156.9. HRMS-ESI (*m*/*z*): calcd for C_16_H_12_O_4_S [M + H]^+^ 335.0145, found: 335.0139.

3-((4-Methylbenzyl)sulfinyl)-2H-chromen-2-one (**5e**)

Yield 41%, white solid, m.p. 174–175 °C. ^1^H-NMR (400 MHz, DMSO-*d*_6_) δ: 2.22 (s, 3H, -CH_3_), 4.04 (d, 1H, *J* = 13.2 Hz, -CH_2_-), 4.48 (d, 1H, *J* = 13.2 Hz, -CH_2_-), 7.02 (d, 1H, *J* = 8.0 Hz, Ar-H), 7.08 (d, 1H, *J* = 8.0 Hz, Ar-H), 7.39 (t, 1H, *J* = 8.0 Hz, Ar-H), 7.52 (d, 1H, *J* = 8.4 Hz, Ar-H), 7.71 (t, 1H, *J* = 8.4 Hz, Ar-H), 7.87 (d, 1H, *J* = 8.0 Hz, Ar-H), 8.05 (s, 1H, =CH-). ^13^C-NMR (101 MHz, DMSO-*d*_6_) δ: 20.5, 56.7, 116.2, 118.1, 125.0, 126.5, 128.7, 129.1, 130.1, 130.9, 133.2, 137.2, 142.3, 153.2, 156.8. HRMS-ESI (*m*/*z*): calcd for C_17_H_14_O_3_S [M + H]^+^ 299.0742, found: 299.0734.

3-((4-Methylbenzyl)sulfonyl)-2H-chromen-2-one (**5f**)

Yield 47%, white solid, m.p. 168–170 °C. ^1^H-NMR (400 MHz, DMSO-*d*_6_) δ: 2.25 (s, 3H, -CH_3_), 4.78 (s, 2H, -CH_2_-), 7.15 (d, 2H, *J* = 8.0 Hz, Ar-H), 7.21 (d, 2H, *J* = 8.0 Hz, Ar-H), 7.46 (t, 1H, *J* = 7.2 Hz, Ar-H), 7.56 (d, 1H, *J* = 8.4 Hz, Ar-H), 7.83 (t, 1H, *J* = 8.4 Hz, Ar-H), 8.01 (d, 1H, *J* = 7.8 Hz, Ar-H), 8.75 (s, 1H, =CH-). ^13^C-NMR (101 MHz, DMSO-*d*_6_) δ: 21.0 57.1, 116.2, 118.0, 125.1, 126.7, 128.9, 129.4, 130.0, 130.9, 133.1, 137.4, 142.5, 153.4, 157.1. HRMS-ESI (*m*/*z*): calcd for C_17_H_14_O_4_S [M + H]^+^ 315.0691, found: 315.0685.

3-((2-Fluorobenzyl)sulfinyl)-6-nitro-2H-chromen-2-one (**5g**)

Yield 40%, yellow, m.p. 190–191 °C. ^1^H-NMR (400 MHz, DMSO-*d*_6_) δ: 4.28 (d, 1H, *J* = 13.2 Hz, -CH_2_-), 4.63 (d, 1H, *J* = 13.2 Hz, -CH_2_-), 7.07 (t, 1H, *J* = 9.2 Hz, Ar-H), 7.19 (t, 1H, *J* = 7.6 Hz, Ar-H), 7.28 (t, 2H, *J* = 7.6 Hz, Ar-H), 7.36 (q, 1H, *J* = 7.6 Hz, Ar-H), 7.79 (d, 1H, *J* = 9.2 Hz, Ar-H), 8.19 (s, 1H, =CH-), 8.51 (d, 1H, *J* = 9.2 Hz, Ar-H), 8.90 (d, 1H, *J* = 2.8 Hz, Ar-H). ^13^C-NMR (101 MHz, DMSO-*d*_6_) δ: 50.2, 115.2 (d, *J* = 22 Hz), 116.2 (d, *J* = 15 Hz), 118.3, 118.8, 124.4 (d, *J* = 4 Hz), 125.2, 128.0, 130.9 (d, *J* = 9 Hz), 133.8, 133.9 (d, *J* = 4 Hz), 141.6, 144.2, 156.5, 157.1, 160.8 (d, *J* = 248 Hz). HRMS-ESI (*m*/*z*): calcd for C_16_H_10_FNO_5_S [M + H]^+^ 348.0342, found: 348.0338.

3-((2-Fluorobenzyl)sulfonyl)-6-nitro-2H-chromen-2-one (**5h**)

Yield 37%, yellow solid, m.p. 257–259 °C. ^1^H-NMR (400 MHz, DMSO-*d*_6_) δ: 4.90 (s, 2H, -CH_2_-), 7.24 (d, 2H, *J* = 7.2 Hz, Ar-H), 7.43 (t, 1H, Ar-H), 7.49 (t, 1H, Ar-H), 7.79 (d, 1H, *J* = 9.2 Hz, Ar-H), 8.60 (d, 1H, *J* = 2.8, 9.2 Hz, Ar-H), 8.98 (s, 1H, =CH-), 9.03 (d, 1H, *J* = 2.8 Hz, Ar-H). ^13^C-NMR (101 MHz, DMSO-*d*_6_) δ: 51.0, 115.7, 116.9, 117.9, 119.1, 124.7, 125.8, 127.8, 131.2, 134.1, 134.5, 141.7, 144.6, 156.6, 157.1, 162.3. HRMS-ESI (*m*/*z*): calcd for C_16_H_10_FNO_6_S [M + H]^+^ 364.0291, 364.0286.

3-((3-Fluorobenzyl)sulfinyl)-6-nitro-2H-chromen-2-one (**5i**)

Yield 39%, yellow solid, m.p. 221–223 °C. ^1^H-NMR (400 MHz, DMSO-*d*_6_) δ: 4.28 (d, 1H, *J* = 13.2 Hz, -CH_2_-), 4.63 (d, 1H, *J* = 13.2 Hz, -CH_2_-), 7.07 (t, 1H, *J* = 9.2 Hz, Ar-H), 7.19 (t, 1H, *J* = 7.2 Hz, Ar-H), 7.28 (t, 2H, *J* = 7.6 Hz, Ar-H), 7.36 (q, 1H, *J* = 7.6 Hz, Ar-H), 7.79 (d, 1H, *J* = 9.2 Hz, Ar-H), 8.19 (s, 1H, =CH-), 8.50 (d, 1H, *J* = 9.2 Hz, Ar-H), 8.90 (d, 1H, *J* = 2.4 Hz, Ar-H). ^13^C-NMR (101 MHz, DMSO-*d*_6_) δ: 56.1, 114.8 (d, *J* = 20 Hz), 117.0 (d, *J* = 22 Hz), 117.8, 118.4, 124.9, 126.4, 127.6, 130.0 (d, *J* = 7 Hz), 131.9 (d, *J* = 7 Hz), 133.3, 141.6, 143.8, 156.0, 156.7, 161.5 (d, *J* = 244 Hz). HRMS-ESI (*m*/*z*): calcd for C_16_H_10_FNO_5_S [M + H]^+^ 348.0342, found: 348.0337.

3-((3-Fluorobenzyl)sulfonyl)-6-nitro-2H-chromen-2-one (**5j**)

Yield 43%, yellow solid, m.p. 232–233 °C. ^1^H-NMR (400 MHz, DMSO-d_6_) δ: 4.89 (s, 2H, -CH_2_-), 7.19–7.26 (q, 3H, Ar-H), 7.39–7.45 (q, 1H, Ar-H), 7.78 (d, 1H, *J* = 9.2 Hz, Ar-H), 8.59 (d, 1H, *J* = 2.8, 9.2 Hz, Ar-H), 9.00 (s, 1H, =CH-), 9.03 (d, 1H, *J* = 2.8 Hz, Ar-H). ^13^C-NMR (101 MHz, DMSO-*d*_6_) δ: 57.0, 115.1, 116.4, 118.0, 118.9, 125.7, 127.0, 127.4, 130.1, 130.8, 133.9, 142.2, 144.6, 156.6, 157.1, 162.3. HRMS-ESI (*m*/*z*): calcd for C_16_H_10_FNO_6_S [M + H]^+^ 364.0291, 364.0285.

3-((4-Fluorobenzyl)sulfinyl)-6-nitro-2H-chromen-2-one (**5k**)

Yield 35%, yellow solid, m.p. 218–220 °C. ^1^H-NMR (400 MHz, DMSO-*d*_6_) δ: 4.15 (d, 1H, *J* = 13.2 Hz, -CH_2_-), 4.60 (d, 1H, *J* = 13.2 Hz, -CH_2_-), 7.14 (t, 2H, *J* = 8.8 Hz, Ar-H), 7.22 (t, 2H, *J* = 7.2 Hz, Ar-H), 7.77 (d, 1H, *J* = 9.2 Hz, Ar-H), 8.26 (s, 1H, =CH-, Ar-H), 8.50 (d, 1H, *J* = 9.2, Ar-H), 8.93 (d, 1H, *J* = 2.8 Hz, Ar-H). ^13^C-NMR (101 MHz, DMSO-*d*_6_) δ: 55.8, 114.9 (d, *J* = 22 Hz), 117.9, 118.5, 124.8, 125.4 (d, *J* = 4 Hz), 127.5, 132.4 (d, *J* = 7 Hz), 133.4, 141.6, 143.8, 156.1, 156.7, 161.8 (d, *J* = 246 Hz). HRMS-ESI (*m*/*z*): calcd for C_16_H_10_FNO_5_S [M + Na]^+^ 370.0161, found: 370.0158.

3-((4-Fluorobenzyl)sulfonyl)-6-nitro-2H-chromen-2-one (**5l**)

Yield 38%, yellow solid, m.p. 258–260 °C. ^1^H-NMR (400 MHz, DMSO-d_6_) δ: 4.85 (s, 2H, -CH_2_-), 7.22 (t, 2H, *J* = 8.8 Hz, Ar-H), 7.42 (t, 2H, *J* = 7.0 Hz, Ar-H), 7.80 (d, 1H, *J* = 9.2 Hz, Ar-H), 8.59 (d, 1H, *J* = 2.4, 9.2 Hz, Ar-H), 8.97 (s, 1H, =CH-), 9.03 (d, 1H, *J* = 2.8 Hz, Ar-H). ^13^C-NMR (101 MHz, DMSO-*d*_6_) δ: 56.8, 115.6, 118.1, 118.4, 125.4, 125.9, 128.7, 133.0, 134.1, 143.2, 150.2, 156.7, 158.1, 162.3. HRMS-ESI (*m*/*z*): calcd for C_16_H_10_FNO_6_S [M + H]^+^ 364.0291, found: 364.0286.

3-((4-Bromobenzyl)sulfinyl)-6-nitro-2H-chromen-2-one (**5m**)

Yield 42%, yellow solid, m.p. 215–216 °C. ^1^H-NMR (400 MHz, DMSO-*d*_6_) δ: 4.15 (d, 1H, *J* = 13.2 Hz, -CH_2_-), 4.60 (d, 1H, *J* = 13.2 Hz, -CH_2_-), 7.14 (d, 2H, *J* = 8.4 Hz, Ar-H), 7.51 (d, 2H, *J* = 8.4 Hz, Ar-H), 7.77 (d, 1H, *J* = 9.2 Hz, Ar-H), 8.28 (s, 1H, =CH-), 8.51 (d, 1H, *J* = 9.2 Hz, Ar-H), 8.94 (d, 1H, *J* = 2.8 Hz, Ar-H). ^13^C-NMR (101 MHz, DMSO-*d*_6_) δ: 55.8, 117.8, 118.4, 121.4, 124.8, 127.5, 128.5, 130.9, 132.4, 133.2, 141.6, 143.7, 155.9, 156.6. HRMS-ESI (*m*/*z*): calcd for C_16_H_10_BrNO_5_S [M + H]^+^ 407.9521, found: 407.9528.

3-((4-Bromobenzyl)sulfonyl)-6-nitro-2H-chromen-2-one (**5n**)

Yield 44%, yellow solid, m.p. 269–271 °C. ^1^H-NMR (400 MHz, DMSO-*d*_6_) δ: 4.85 (s, 2H, -CH_2_-), 7.32 (d, 2H, *J* = 8.0 Hz, Ar-H), 7.58 (d, 2H, *J* = 8.0 Hz, Ar-H), 7.78 (d, 1H, *J* = 9.2 Hz, Ar-H), 8.59 (d, 1H, *J* = 2.8, 9.2 Hz, Ar-H), 8.98 (s, 1H, =CH-), 9.03 (d, 1H, *J* = 2.8 Hz, Ar-H). ^13^C-NMR (101 MHz, DMSO-*d*_6_) δ: 56.9, 117.3, 118.4, 126.4, 129.3, 129.5, 130.4, 131.0, 131.7, 132.0, 134.1, 143.7, 156.1, 158.9. HRMS-ESI (*m*/*z*): calcd for C_16_H_10_BrNO_6_S [M + H]^+^ 423.9490, found: 423.9483.

3-((3-Methoxybenzyl)sulfinyl)-6-nitro-2H-chromen-2-one (**5o**)

Yield 36%, yellow solid, m.p. 213–215 °C. ^1^H-NMR (400 MHz, DMSO-*d*_6_) δ: 3.67 (s, 3H, -OCH_3_), 4.09 (d, 1H, *J* = 13.2 Hz, -CH_2_-), 4.55 (d, 1H, *J* = 13.2 Hz, -CH_2_-), 6.73 (s, 1H, Ar-H), 6.76 (d, 1H, *J* = 2.0 Hz, Ar-H), 6.86 (d, 1H, *J* = 8.0 Hz, Ar-H), 7.21 (t, 1H, *J* = 8.0 Hz, Ar-H), 7.77 (d, 1H, *J* = 9.2 Hz, Ar-H), 8.35 (s, 1H, =CH-), 8.49 (d, 1H, *J* = 9.2 Hz, Ar-H), 8.94 (d, 1H, *J* = 2.8 Hz, Ar-H). ^13^C-NMR (101 MHz, DMSO-*d*_6_) δ: 54.8, 57.3, 113.6, 115.6, 117.8, 118.5, 122.3, 124.8, 127.5, 129.2, 131.0, 133.7, 141.3, 143.7, 156.1, 156.7, 158.8. HRMS-ESI (*m*/*z*): calcd for C_17_H_13_NO_6_S [M + H]^+^ 360.0542, found: 360.0536.

3-((3-Methoxybenzyl)sulfonyl)-6-nitro-2H-chromen-2-one (**5p**)

Yield 40%, yellow solid, m.p. 238–240 °C. ^1^H-NMR (400 MHz, DMSO-*d*_6_) δ: 3.72 (s, 3H, -OCH_3_), 4.81 (s, 2H, -CH_2_-), 6.89–6.95 (m, 3H, Ar-H), 7.27 (t, 1H, *J* = 8.0 Hz, Ar-H), 7.78 (d, 1H, *J* = 8.8 Hz, Ar-H), 8.59 (d, 1H, *J* = 2.4, 8.8 Hz, Ar-H), 8.99 (s, 1H, =CH-), 9.03 (d, 1H, *J* = 2.4 Hz, Ar-H). ^13^C-NMR (101 MHz, DMSO-*d*_6_) δ: 55.4, 57.9, 113.7, 115.2, 118.5, 118.8, 123.0, 126.1, 129.3, 130.4, 131.8, 135.1, 142.7, 145.3, 156.6, 156.9, 159.9. HRMS-ESI (*m*/*z*): calcd for C_17_H_13_NO_7_S [M + H]^+^ 376.0491, found: 376.0484.

3-((4-Methylbenzyl)sulfinyl)-6-nitro-2H-chromen-2-one (**5q**)

Yield 34%, yellow solid, m.p. 234–245 °C. ^1^H-NMR (400 MHz, DMSO-*d*_6_) δ: 2.24 (s, 3H, -CH_3_), 4.07 (d, 1H, *J* = 13.2 Hz, -CH_2_-), 4.54 (d, 1H, *J* = 13.2 Hz, -CH_2_-), 7.07 (d, 2H, *J* = 8.0 Hz, Ar-H), 7.12 (d, 2H, *J* = 8.0 Hz, Ar-H), 7.77 (d, 1H, *J* = 9.2 Hz, Ar-H), 8.33 (s, 1H, =CH-), 8.50 (d, 1H, *J* = 9.2 Hz, Ar-H), 8.94 (d, 1H, *J* = 2.4 Hz, Ar-H). ^13^C-NMR (101 MHz, DMSO-*d*_6_) δ: 21.1, 57.5, 118.4, 119.0, 125.4, 126.9, 128.0, 129.3, 130.7, 134.2, 137.9, 141.9, 144.3, 156.6, 157.2. HRMS-ESI (*m*/*z*): calcd for C_17_H_13_NO_5_S [M + Na]^+^ 366.0412, found: 366.0406.

3-((4-Methylbenzyl)sulfonyl)-6-nitro-2H-chromen-2-one (**5r**)

Yield 37%, yellow solid; m.p. 247–249 °C. ^1^H-NMR (400 MHz, DMSO-*d*_6_) δ: 2.27 (s, 3H, -CH_3_), 4.79 (s, 2H, -CH_2_-), 7.17 (d, 2H, *J* = 8.0 Hz, Ar-H), 7.23 (d, 2H, *J* = 8.0 Hz, Ar-H), 7.78 (d, 1H, *J* = 9.2 Hz, Ar-H), 8.58 (d, 1H, *J* = 2.8, 9.2 Hz, Ar-H), 8.96 (d, 1H, =CH-), 9.02 (d, 1H, *J* = 2.8 Hz, Ar-H). ^13^C-NMR (101 MHz, DMSO-*d*_6_) δ: 21.5, 58.7, 118.7, 119.9, 125.9, 127.6, 129.0, 130.2, 130.9, 135.6, 138.0, 142.2, 144.1, 156.1, 157.3. HRMS-ESI (*m*/*z*): calcd for C_17_H_13_NO_6_S [M + H]^+^ 360.0542, found: 360.0536.

3-((2-Chlorobenzyl)sulfonyl)-7-methoxy-2H-chromen-2-one (**5s**)

Yield 49%, white solid, m.p. 206–208 °C. ^1^H-NMR (400 MHz, DMSO-*d*_6_) δ: 3.92 (s, 3H, -OCH_3_), 4.95 (s, 2H, -CH_2_-), 7.07 (d, *J* = 8.8 Hz, 1H, Ar-H), 7.18 (d, *J* = 2.4 Hz, 1H, Ar-H), 7.39 (m, 2H, Ar-H), 7.35–7.52 (m, 2H, Ar-H), 7.92 (d, *J* = 8.8 Hz, 1H, Ar-H), 8.63 (s, 1H, =CH-). ^13^C NMR (101 MHz, DMSO-d_6_) δ: 56.2, 57.0, 101.2, 111.4, 114.5, 121.7, 126.8, 128.0, 130.1, 131.2, 133.1, 134.5, 134.9, 150.0, 156.6, 157.9, 166.2. HRMS-ESI (*m*/*z*): calcd for C_17_H_13_ClO_5_S [M + H]^+^ 365.0250, found: 365.0245.

3-((2-Chlorobenzyl)sulfonyl)-8-methoxy-2H-chromen-2-one (**5t**)

Yield 45%, white solid, m.p. 258–260 °C. ^1^H-NMR (400 MHz, DMSO-*d*_6_) δ: 3.95 (s, 3H, -OCH_3_), 4.99 (s, 2H, -CH_2_-), 7.38 (d, 1H, *J* = 2.0 Hz, Ar-H), 7.40 (m, 2H, Ar-H), 7.48 (dd, *J*_1_ = 8.0 Hz, *J_2_* = 2.0 Hz, 1H, Ar-H), 7.52 (d, *J* = 2.8 Hz, 1H, Ar-H), 7.55 (d, *J* = 8.4 Hz, 2H, Ar-H), 8.73 (s, 1H, =CH-). ^13^C NMR (101 MHz, DMSO-d_6_) δ: 56.3, 56.8, 118.2, 118.4, 122.5, 126.0, 126.2, 126.5, 128.0, 130.2, 131.3, 134.5, 135.0, 144.6, 147.0, 150.3, 156.0. HRMS-ESI (*m*/*z*): calcd for C_17_H_13_ClO_5_S [M + H]^+^ 365.0250, found: 365.0244.

3-((4-Bromobenzyl)sulfonyl)-8-methoxy-2H-chromen-2-one (**5u**)

Yield 54%, white solid, m.p. 267–269 °C. ^1^H-NMR (400 MHz, DMSO-*d*_6_) δ: 3.70 (s, 3H, -OCH_3_), 3.92 (s, 6H, -OCH_3_ × 2), 4.73 (s, 2H, -CH_2_-), 6.60 (d, *J* = 2.0 Hz, 1H, Ar-H), 6.78 (d, *J* = 2.0 Hz, 1H, Ar-H), 6.84 (s, 1H, Ar-H), 6.86 (d, *J* = 2.0 Hz, 1H, Ar-H), 6.91 (d, *J* = 8.4 Hz, 1H, Ar-H), 7.25 (t, *J* = 8.0 Hz, 1H, Ar-H), 8.36 (s, 1H, =CH-). ^13^C-NMR (101 MHz, DMSO-*d*_6_) δ: 56.1, 57.6, 117.4, 117.5, 121.8, 122.1, 125.0, 125.2, 127.0, 131.4, 133.0, 143.8, 146.2, 150.0, 155.3. HRMS-ESI (*m*/*z*): calcd for C_17_H_13_BrO_5_S [M + H]^+^ 408.9745, found: 408.9740.

3-((2-Bromobenzyl)sulfonyl)-8-methoxy-2H-chromen-2-one (**5v**)

Yield 57%, white solid, m.p. 252–254 °C. ^1^H-NMR (400 MHz, DMSO-*d*_6_) δ: 3.96 (s, 3H, -OCH_3_), 5.00 (s, 2H, -CH_2_-), 7.32 (t, *J* = 8.0 Hz, 1H, Ar-H), 7.39 (d, *J* = 8.0 Hz, 1H, Ar-H), 7.43 (d, *J* = 8.0 Hz, 1H, Ar-H), 7.51–7.57 (q, 3H, Ar-H), 7.65 (d, *J* = 8.0 Hz, 1H, Ar-H), 8.74 (s, 1H, =CH-). ^13^C-NMR (101 MHz, DMSO-*d*_6_) δ: 56.8, 58.6, 118.1, 118.3, 122.4, 125.7, 125.8, 126.2, 128.2, 128.3, 131.3, 133.4, 134.4, 144.6, 146.8, 150.3, 155.9. HRMS-ESI (*m*/z): calcd for C_17_H_13_BrO_5_S [M + H]^+^ 408.9745, found: 408.9743.

### 3.3. Biological Evaluation

#### 3.3.1. Cell Culture and Irradiation

Human umbilical vein endothelial cells (HUVECs) between passages 3 and 6 served as the in vitro model in this study [16]. The cells were maintained under sterile conditions in Roswell Park Memorial Institute-1640 (RPMI-1640) medium containing 10% (*v*/*v*) fetal bovine serum (FBS), along with penicillin (100 U/mL) and streptomycin (100 μg/mL) They were maintained at 37 °C in a humidified incubator with 5% CO_2_, and the medium pH was adjusted to 7.2. When the cells reached approximately 80% confluence, they were detached with 0.25% trypsin–EDTA and resuspended in fresh medium to a standardized density for subsequent experiments.

Cell proliferation was evaluated using the MTS tetrazolium assay (Promega, Beijing, China) according to the manufacturer’s protocol. Briefly, HUVEC monolayers seeded in 96-well plates were treated with the target compounds for 24 h before irradiation. During irradiation, the plates were covered with individual Plexiglas sheets and exposed to γ-radiation at prescribed doses: 8.0 Gy for cell survival analysis and 6.0 Gy for the comet assay. All irradiations were conducted using a ^60^Co γ-ray source at the Beijing Institute of Radiation Medicine, Beijing, China.

#### 3.3.2. Cell Survival

Cell viability after irradiation was evaluated using the conventional MTS tetrazolium assay. Briefly, cells were seeded in 96-well plates at 4000 cells per mL, with 100 μL of culture medium added to each well. The test compounds were first dissolved in DMSO to prepare a 100 mmol/L stock solution, which was subsequently diluted in culture medium to achieve the final concentrations (20 μmol/L or 40 μmol/L). Following a 24 h incubation period, the cells were treated with the synthesized compounds for another 24 h and then subjected to ^60^Co γ-irradiation (8.0 Gy). After irradiation, the cells were cultured for 4 days; during this time, the medium was replaced with fresh medium (100 μL per well) every two days. At the end of this incubation, the medium was renewed once more, and 10 μL of MTS reagent was introduced into each well.

The plate was subsequently maintained at 37 °C in a 5% CO_2_ atmosphere for 3 h. Untreated and non-irradiated cells were designated as the negative control. A microplate reader was used to measure the absorbance at 492 nm, and the cell survival rates were determined using the following formula:

Survival rate (%) = (OD_sample_ − OD_blank_)/(OD_negative control_ − OD_blank_) × 100%

All reported survival values represent the mean of three independent experiments. Statistical analysis and graph plotting were performed using GraphPad Prism software.

#### 3.3.3. Single-Cell Electrophoresis (Comet Assay)

In fluorescence microscopy, a characteristic comet-like tail forms as damaged cellular DNA (comprising strand breaks and fragments) migrates away from the intact DNA under an electric field [31].

In this research, the alkaline comet assay was employed to measure DNA damage, aiming to investigate the radioprotective efficacy of the target compounds. The comet assay was conducted using the OxiSelect™ kit (Cell Biolabs, San Diego, CA, USA), following the manufacturer’s protocol. Following irradiation (6.0 Gy), control and treated cells were collected at the 24 h time point and resuspended to a final density of 1 × 10^5^ mL^−1^. The cell suspension was mixed with comet agarose at a 1:10 (*v*/*v*) ratio. Then, 75 μL of the mixture was promptly pipetted onto each well of a comet slide. Slides were kept at 4 °C in the dark for 15 min to allow the agarose to solidify. Following solidification, slides were horizontally immersed in pre-chilled lysis buffer and incubated at 4 °C in the dark for 30 min. This was followed by equilibration in an alkaline unwinding solution (300 mmol/L NaOH, 1 mmol/L EDTA, pH > 13) at 4 °C for another 30 min. Electrophoresis was performed in the same alkaline buffer at 1.0 V cm^−1^ (300 mA) for 20 min using a horizontal electrophoresis unit.

Following electrophoresis, the slides were processed through fixation in 70% ethanol (5 min), air-drying (1–2 h), and staining with Vista Green DNA dye (1:10,000 dilution in the supplied buffer; 5 min). Comet visualization was performed at 400× magnification using an epifluorescence microscope with excitation/emission at 494/521 nm. For each slide, 70 comets were randomly selected and captured. DNA damage was quantified by measuring tail length (defined as the distance of DNA migration from the nucleus in micrometers), which directly reflects the extent of DNA fragmentation. Tail length measurements were performed using the Comet Assay Software Project (CASP 1.2.3 beta 2). The entire experiment was repeated independently three times.

#### 3.3.4. Mice and Irradiation

This study utilized male C57/BL mice (18–22 g) sourced from SPF (Beijing) Biotechnology Co., Ltd. (Beijing, China), and all animals were housed at the Laboratory Animal Center of the Beijing Institute of Radiation Medicine, with five mice per cage. The housing environment was maintained at 25 °C and 50% ± 10% relative humidity under a 12 h light/dark cycle (lights on from 08:00 to 20:00). Fresh air was supplied at a rate of 10–15 cycles per hour. Following one week of acclimatization, mice were irradiated with ^60^Co γ-rays at 0.62 Gy min^−1^ while housed in well-ventilated Lucite boxes. After irradiation, animals were returned to their home cages for a 30-day survival observation. This animal study was approved by the Animal Ethics Committee of the Beijing Institute of Radiation Medicine (approval number: IACUC of AMMS 2020-712) and conducted in accordance with the relevant guidelines.

#### 3.3.5. Survival Study in Mice

Mice were randomly assigned to four groups (n = 10 per group): an irradiated vehicle control group (Vehicle), and three irradiated treatment groups receiving Ex-RAD, compound **5q**, or compound **5u**, respectively. Compounds were formulated in a vehicle consisting of 20% hydroxypropyl-β-cyclodextrin (HPCD) in normal saline.

The treatment protocols for each group were as follows. For the Vehicle, **5q**, and **5u** groups, mice were given two intraperitoneal injections (0.2 mL, 300 mg/kg of the respective agent) at 24 h and 15 min before irradiation. For the Ex-RAD group, mice received two subcutaneous injections (0.2 mL, 300 mg/kg) at the same pre-irradiation time points.

All animals were subjected to whole-body irradiation at a dose of 8.0 Gy ^60^Co γ-irradiation. Survival and body weight changes were recorded daily for 30 days following radiation exposure.

### 3.4. Statistical Analysis

For statistical analysis, data are presented as the mean ± standard deviation. Differences between two groups in cell survival, comet assay outcomes, and body weight were evaluated using a two-tailed paired Student’s t-test, with statistical significance defined as *p* < 0.05. For survival data, analysis was performed using the Kaplan–Meier method, with curve comparisons made via the log-rank test; Fisher’s exact test was used to assess differences in 30-day survival rates. All analyses were conducted in GraphPad Prism (Version 5.01, GraphPad Software, San Diego, CA, USA).

## 4. Conclusions

In this study, a series of 22 compounds derived from EX-RAD were designed utilizing bioisosterism as the core strategy. All target compounds were synthesized and their structures were verified by ^1^H NMR, ^13^C NMR and HRMS. In vitro assays demonstrated that compounds **5k**, **5q**, **5s**, **5u** significantly improved irradiated cell viability relative to Ex-RAD. Comet assay results further indicated that pretreatment with compound **5q** or **5u** markedly reduced tail lengths and Olive tail moment values in HUVECs, demonstrating a pronounced protective effect against DNA damage. Furthermore, in an established murine model, treatment with compound **5u** resulted in a substantially higher thirty-day survival rate (90%) compared to the vehicle (20%) and Ex-RAD (50%) groups, underscoring its potential as a promising radioprotective candidate.

Overall, these findings validate the feasibility of our bioisosterism-based design strategy. The improved survival rate observed in mice may be linked to the attenuation of DNA damage, but the specific mechanism remains unclear and warrants further investigation.

## Data Availability

The data presented in this study are available on request from the corresponding author.

## Supplementary Materials

The following supporting information can be downloaded at: https://www.mdpi.com/article/10.3390/molecules31030487/s1, Supplementary data for this article are available online: the synthetic routes and analytical data for the intermediates mentioned in Scheme 1; ^1^H-NMR, ^13^C-NMR and HRMS-ESI spectra of some target compounds; Clear and representative fluorescence microscopy images of HUVECs 24 h after 6.0 Gy irradiation [22,23,32,33,34,35,36,37,38].
