# Supplementary material for: Synthesis and Radioprotective Activity of Benzyl Sulfoxide/Sulfone Coumarins Derived from Ex-RAD
Source: Molecules. 2026 Jan 30;31(3):487. doi: 10.3390/molecules31030487 (PMC12899614; doi:10.3390/molecules31030487)

Synthesis and Radioprotective Activity of Benzyl Sulfoxide/Sulfone Coumarins Derived from Ex-RAD

Tao Wang[1, 2,*], Chunrui Zhou[1], Ningfan Liu[1], Tao Peng[2], Lin Wang[2,*], and Shouguo Zhang[2,*]

[1] School of Chemistry and Chemical Engineering, Henan Institute of Science and Technology, Xinxiang, 453003, P. R. China

[2] Beijing Institute of Radiation Medicine, Beijing 100850, P. R. China

Supplementary Materials

1. Synthesis of Intermediates

1.1. General procedure for the synthesis of compounds **2a**–**2j**

To an ice-cold solution of sodium hydroxide (2.2 eq.) in methanol was added mercaptoacetic acid (1.1 eq.) and substituted benzyl chloride/bromide (**1a**–**1j**, 1.0 eq.). When the reaction was completed (1–2 h), the mixture was evaporated to dryness and dissolved in water. Dilute hydrochloric acid was added until the precipiate formed, which was filtered, washed with water and dried under vacuum to give title compounds **2a**–**2j**.

2-(Benzylthio)acetic acid (**2a**). Condensation of benzyl chloride (**1a**) with mercaptoacetic acid gave the corresponding product. White solid, yield 58%; m.p. 63–65 ºC. It was identified by comparing its physical data with the literature data [1].

2-(2-Chlorobenzylthio)acetic acid (**2b**). Condensation of 2-chlorobenzyl chloride (**1b**) with mercaptoacetic acid gave the corresponding product. White solid, yield 85%; mp 52–54 ºC. It was identified by comparing its physical data with the literature data [2].

2-(3-Chlorobenzylthio)acetic acid (**2c**). Condensation of 3-chlorobenzyl chloride (**1c**) with mercaptoacetic acid gave the corresponding product. White solid, yield 87%; mp 62–63 ºC. It was identified by comparing its physical data with the literature data [1].

2-(2-Fluorobenzylthio)acetic acid (**2d**). Condensation of 2-fluorobenzyl chloride (**1d**) with mercaptoacetic acid gave the corresponding product. Yellow oil, yield 89%. It was identified by comparing its physical data with the literature data [1].

2-(3-Fluorobenzylthio)acetic acid (**2e**). Condensation of 3-fluorobenzyl chloride (**1e**) with mercaptoacetic acid gave the corresponding product. Yellow oil, yield 87%. It was identified by comparing its physical data with the literature data [3].

2-(4-Fluorobenzylthio)acetic acid (**2f**). Condensation of 4-fluorobenzyl chloride (**1f**) with mercaptoacetic acid gave the corresponding product. Yellow oil, yield 70%. It was identified by comparing its physical data with the literature data [3].

2-(2-Bromobenzylthio)acetic acid (**2g**). Condensation of 2-bromobenzyl bromide (**1g**) with mercaptoacetic acid gave the corresponding product. White solid, yield 90%; mp 50–52 ºC. It was identified by comparing its physical data with the literature data [4].

2-(4-Bromobenzylthio)acetic acid (**2h**). Condensation of 4-bromobenzyl bromide (**1h**) with mercaptoacetic acid gave the corresponding product. White solid, yield 80%; mp 74–76 ºC. It was identified by comparing its physical data with the literature data [3].

2-(3-Methoxybenzylthio)acetic acid (**2i**). Condensation of 3-methoxybenzyl chloride (**1i**) with mercaptoacetic acid gave the corresponding product. Pale yellow oil, yield 87%. It was identified by comparing its physical data with the literature data [5].

2-(4-Methylbenzylthio)acetic acid (**2j**). Condensation of 4-methylbenzyl chloride (**1j**) with mercaptoacetic acid gave the corresponding product. Pale yellow solid, yield 79%; mp 65–67 ºC. It was identified by comparing its physical data with the literature data [6].

1.2. General procedure for the synthesis of compounds **3a**–**3j**

To an ice-cold sodium hydroxide aqueous solution was added substituted benzylthio acetic acid (**2a**–**2j**, 1.0 eq.) and 30% hydrogen peroxide (2.0 eq.). When the reaction was completed (2 h), dilute hydrochloric acid was added until the precipiate formed, which was filtered, washed with water and dried under vacuum to give title compounds **3a**–**3j**.

2-(Benzylsulfinyl)acetic acid (**3a**). Oxidation of **2a** with 30% hydrigen peroxide gave the product. White solid, yield 65%; m.p. 129–131 ºC. It was identified by comparing its physical data with the literature data [7].

2-((2-Chlorobenzyl)sulfinyl)acetic acid (**3b**). Oxidation of **2b** with 30% hydrigen peroxide gave the product. White solid, yield 81%; m.p. 141–143 ºC. It was identified by comparing its physical data with the literature data [7].

2-((3-Chlorobenzyl)sulfinyl)acetic acid (**3c**). Oxidation of **2c** with 30% hydrigen peroxide gave the product. White solid, yield 84%; m.p. 173–174 ºC. It was identified by comparing its physical data with the literature data [7].

2-((2-Fluorobenzyl)sulfinyl)acetic acid (**3d**). Oxidation of **2d** with 30% hydrigen peroxide gave the product. White solid, yield 81%; m.p. 123–125 ºC. It was identified by comparing its physical data with the literature data [8].

2-((3-Fluorobenzyl)sulfinyl)acetic acid (**3e**). Oxidation of **2e** with 30% hydrigen peroxide gave the product. White solid, yield 87%; m.p. 144–146 ºC. It was identified by comparing its physical data with the literature data [8].

2-((4-Fluorobenzyl)sulfinyl)acetic acid (**3f**). Oxidation of **2f** with 30% hydrigen peroxide gave the product. White solid, yield 84%; m.p. 112–114 ºC. It was identified by comparing its physical data with the literature data [2].

2-((2-Bromobenzyl)sulfinyl)acetic acid (**3g**). Oxidation of **2g** with 30% hydrigen peroxide gave the product. White solid, yield 85%; m.p. 130–131 ºC. It was identified by comparing its physical data with the literature data [4].

2-((4-Bromobenzyl)sulfinyl)acetic acid (**3h**). Oxidation of **2h** with 30% hydrigen peroxide gave the product. White solid, yield 88%; m.p. 147–149 ºC. It was identified by comparing its physical data with the literature data [9].

2-((3-Methoxybenzyl)sulfinyl)acetic acid (**3i**). Oxidation of **2i** with 30% hydrigen peroxide gave the product. White solid, yield 66%; m.p. 113–115 ºC. It was identified by comparing its physical data with the literature data [5].

2-((4-Methylbenzyl)sulfinyl)acetic acid **(3j)**. Oxidation of **2j** with 30% hydrigen peroxide gave the product. White solid, yield 89%; m.p. 140–141 ºC. It was identified by comparing its physical data with the literature data [5].

1.3. General procedure for the synthesis of compounds **3k-3t**

To an ice-cold solution of substituted benzylthio acetic acid (**2a-2j**, 1.0 eq.) in glacial acetic acid was added 30% hydrogen peroxide (4.0 eq.) slowly. 15 minutes later, the reaction mixture was heated at 50 ºC for 4 h. The mixture was then poured into cold water, and the precipitate was collected by filtration, washed with water and dried in vacuo to give **3k-3t**.

2-(Benzylsulfonyl)acetic acid (**3k**). Oxidation of **2a** with 30% hydrigen peroxide gave the product. White solid, yield 93%; m.p. 137–139 ºC. It was identified by comparing its physical data with the literature data [7].

2-((2-Chlorobenzyl)sulfonyl)acetic acid (**3l**). Oxidation of **2b** with 30% hydrigen peroxide gave the product. White solid, yield 80%; m.p. 163–165 ºC. It was identified by comparing its physical data with the literature data [8].

2-((3-Chlorobenzyl)sulfonyl)acetic acid (**3m**). Oxidation of **2c** with 30% hydrigen peroxide gave the product. White solid, yield 90%; m.p. 161–163 ºC. It was identified by comparing its physical data with the literature data [7].

2-((2-Fluorobenzyl)sulfonyl)acetic acid (**3n**). Oxidation of **2d** with 30% hydrigen peroxide gave the product. White solid, yield 71%; m.p. 134–136 ºC. It was identified by comparing its physical data with the literature data [8].

2-((3-Fluorobenzyl)sulfonyl)acetic acid (**3o**). Oxidation of **2e** with 30% hydrigen peroxide gave the product. White solid, yield 75%; m.p. 126–128 ºC. It was identified by comparing its physical data with the literature data [8].

2-((4-Fluorobenzyl)sulfonyl)acetic acid (**3p**). Oxidation of **2f** with 30% hydrigen peroxide gave the product. White solid, yield 71%; m.p. 160–163 ºC. It was identified by comparing its physical data with the literature data [2].

2-((2-Bromobenzyl)sulfonyl)acetic acid (**3q**). Oxidation of **2g** with 30% hydrigen peroxide gave the product. White solid, yield 79%; m.p. 175–177 ºC. It was identified by comparing its physical data with the literature data [4].

2-((4-Bromobenzyl)sulfonyl)acetic acid (**3r**). Oxidation of **2h** with 30% hydrigen peroxide gave the product. White solid, yield 88%; m.p. 177–179 ºC. It was identified by comparing its physical data with the literature data [9].

2-((3-Methoxybenzyl)sulfonyl)acetic acid (**3s**). Oxidation of **2i** with 30% hydrigen peroxide gave the product. White solid, yield 70%; m.p. 133–135 ºC. It was identified by comparing its physical data with the literature data [5].

2-((4-Methylbenzyl)sulfonyl)acetic acid **(3t).** Oxidation of **2j** with 30% hydrigen peroxide gave the product. White solid, yield 73%; m.p. 145–146 ºC. It was identified by comparing its physical data with the literature data [5].

References

[1] Kojima, K; Koyama, K; Kurata, H; Fukumi, H; Tabata, K; Yasuda, H. Preparation of disubstituted thiophenes and ulcer inhibitors. *Jpn. Kokai Tokkyo Koho*, **1996**, JP 08127581, A 19960521.

[2] Janczewski, M.; Ksiezopolski, J. Effect of molecular structure on optical properties of sulfoxide systerms, m-Bromobenzylsulfinylacetic acids and some if their derivatives, Part VII. *Pol. J. Chem.* **1984**, *58*, 103-106.

[3] Zhou, N.; Feng, T.; Shen, X.; Cui, J.; Wu, R.; Wang, L.; Chen, H. Synthesis, characterization, and radioprotective activity of α,β-unsaturated aryl sulfone analogs and their Tempol conjugates. *MedChemComm*. **2017**, *8*, 1063-1068.

[4] Abraham, D.J.; Kennedy, P.E.; Mehanna, A.S.; Patwa, D.C.; Williams, F.L. Design, synthesis, and testing of potential antisickling agents. 4. structure activity relationships of benzyloxy and phenoxy acids. *J. Med. Chem*. **1984**, *16*, 967-981.

[5] Janczewski, M.; Janowski, W. Effect of molecular structure on optical properties of sulfoxide systems. Optical relations in the group of isomeric o-, m-, and p-methylbenzylsulfinylacetic acids. *Roczniki Chemii*. **1975**, *49*, 1961-1962.

[6] Bordwell, F.G.; Wolfinger, M.D.; O'Dwyer, J.B. Synthesis of dihalomethyl and alpha-haloalkyl sulfones by the halogenative decarboxylation of alpha-aryl- and alpha-alkylsulfonylalkane carboxylic acids. *ChemInform*. 1974, *5*, 2516-2519.

[7] Mccaw, P.G.; Buckley N.M.; Eccles K.S.; Lawrence S.E.; Maguire A.R.; Collins S.G. Synthesis of Cyclic α-Diazo-β-keto Sulfoxides in Batch and Continuous Flow. *J. Org. Chem.* **2017**, *82*, 3666-3679.

[8] Ning X.L.; Guo Y.; Wang X.W.; Ma X.Y.; Tian C.; Shi X.Q.; Zhu R.Z.; Cheng C.; Du Y.S.; Ma Z.Z.; et al. Design, synthesis, and biological evaluation of (E)-3,4-dihydroxystyryl aralkyl sulfones and sulfoxides as novel multifunctional neuroprotective Agents. *J. Med. Chem*. **2014**, *57*, 4302-4312.

[9] Janczewski, M.; Ksiezopolski, J. Effect of molecular structure on optical properties of sulfoxide systerms, p-Bromobenzylsulfinylacetic acids and some if their derivatives, Part III. *Pol. J. Chem.*

1981, *55*, 535-546.

2. Some copies of ^{1}H-NMR, ^{13}C-NMR and HRMS for the target compounds

^{1}H-NMR of Compound **5a**:

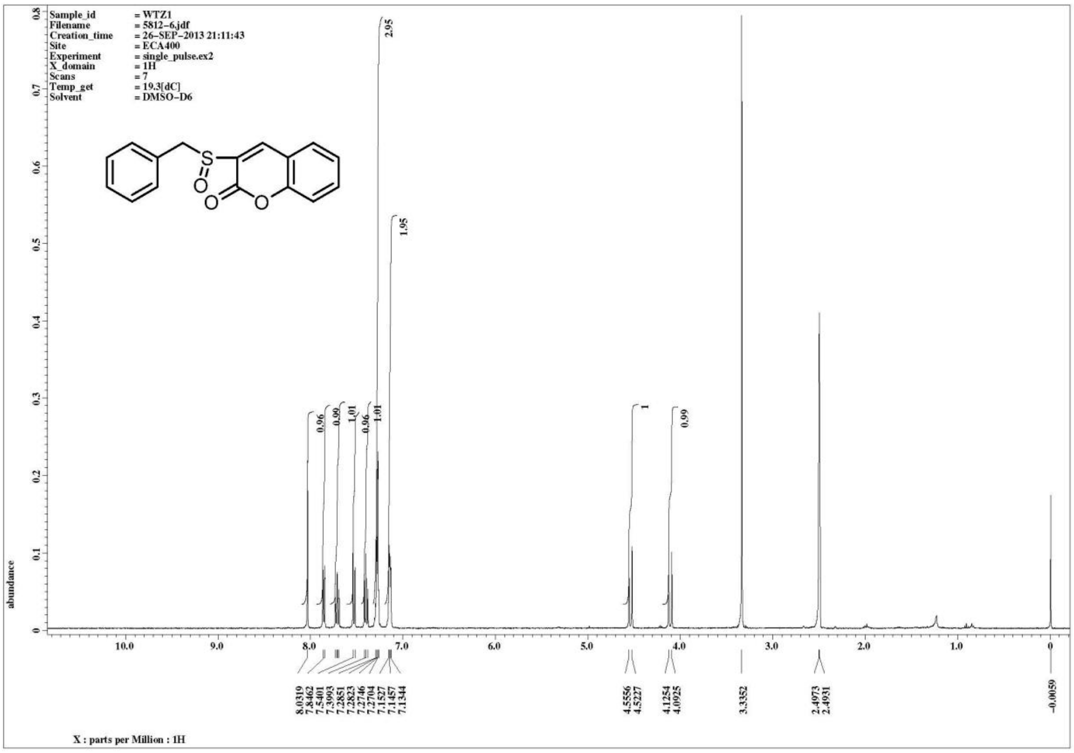

^{13}C-NMR of Compound **5a**:

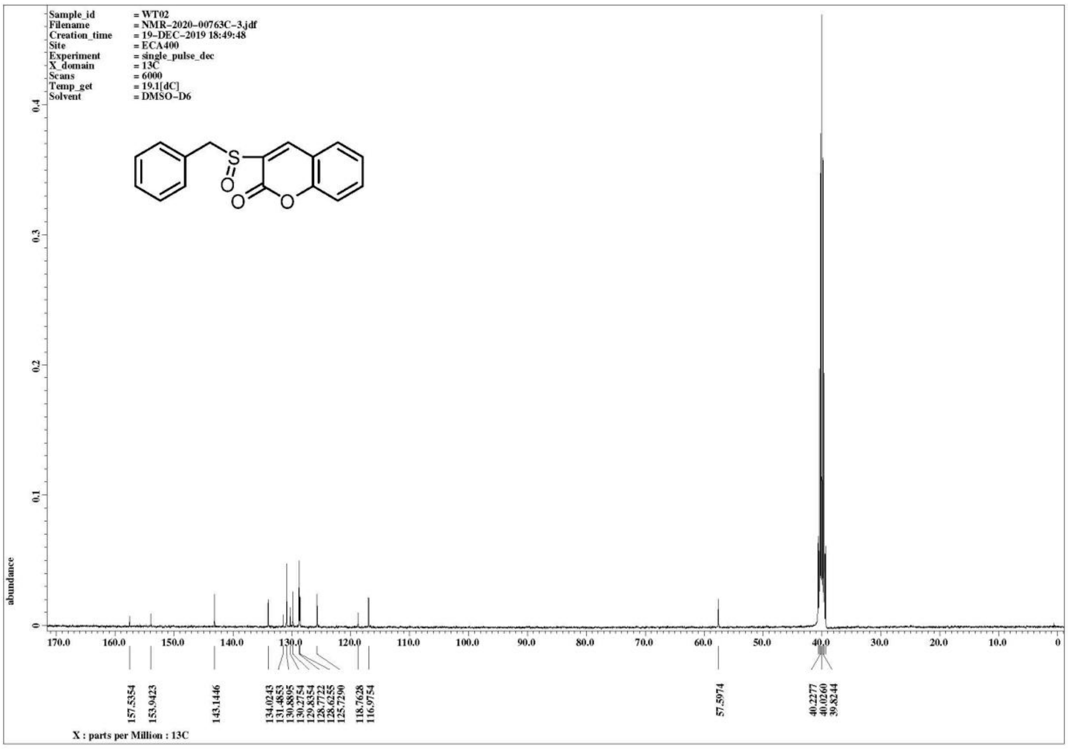

HRMS of Compound **5a**:

Qualitative Analysis Report

**Data Filename**	0586.d	**Sample Name**	FS1
**Instrument Name**	TOF G6230A	**Acquired Time**	2019-12-09
**Acq Method**	YCL.M	**Acquired SW**	6200 series TOF/6500 series
**IRM Calibration Status**	**Success**		
**User Chormatograms**			

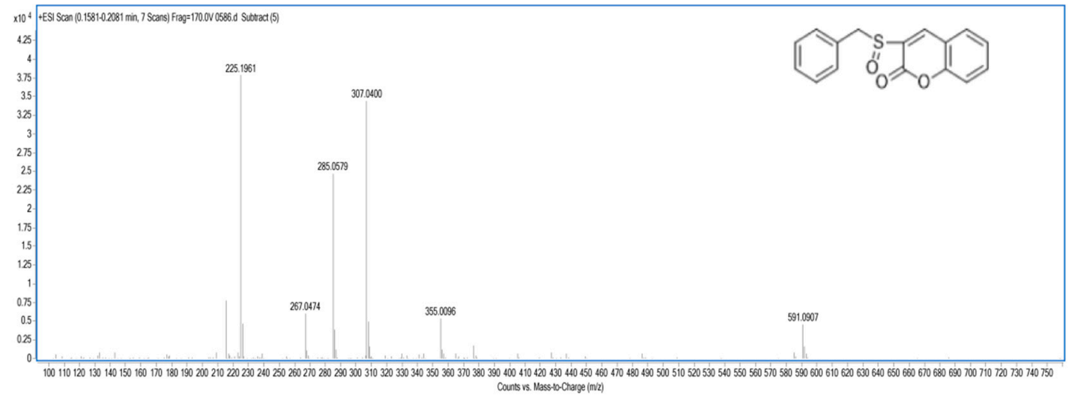

^{1}H-NMR of Compound **5b**:

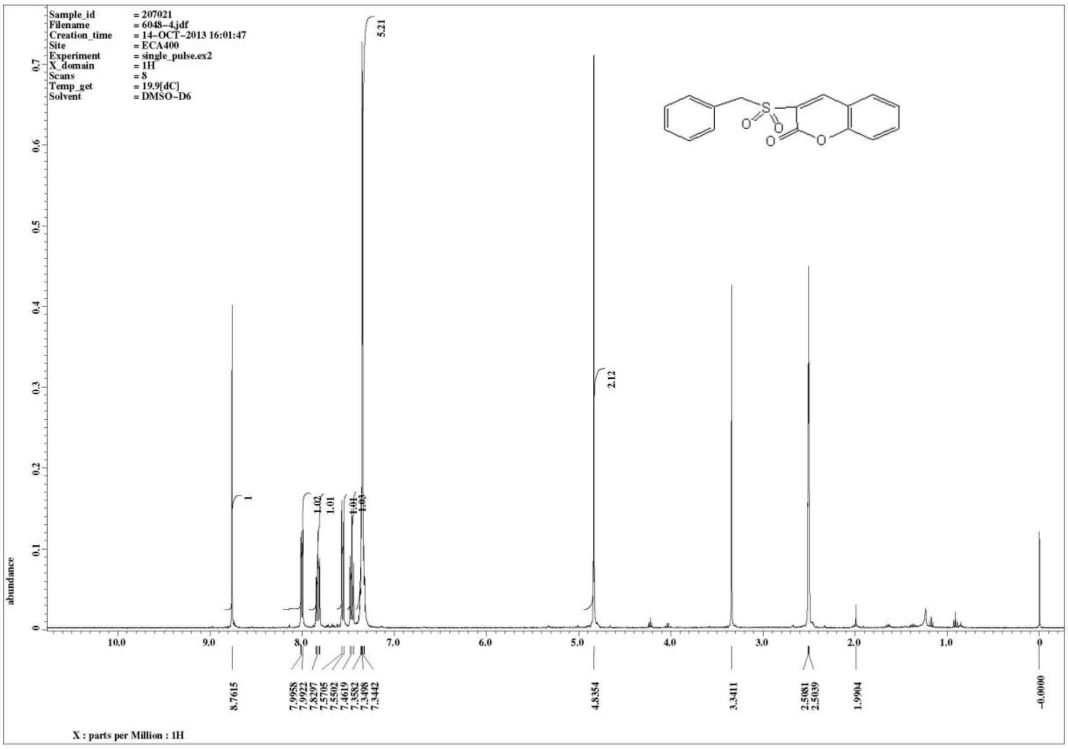

^{1}H-NMR of Compound **5c**:

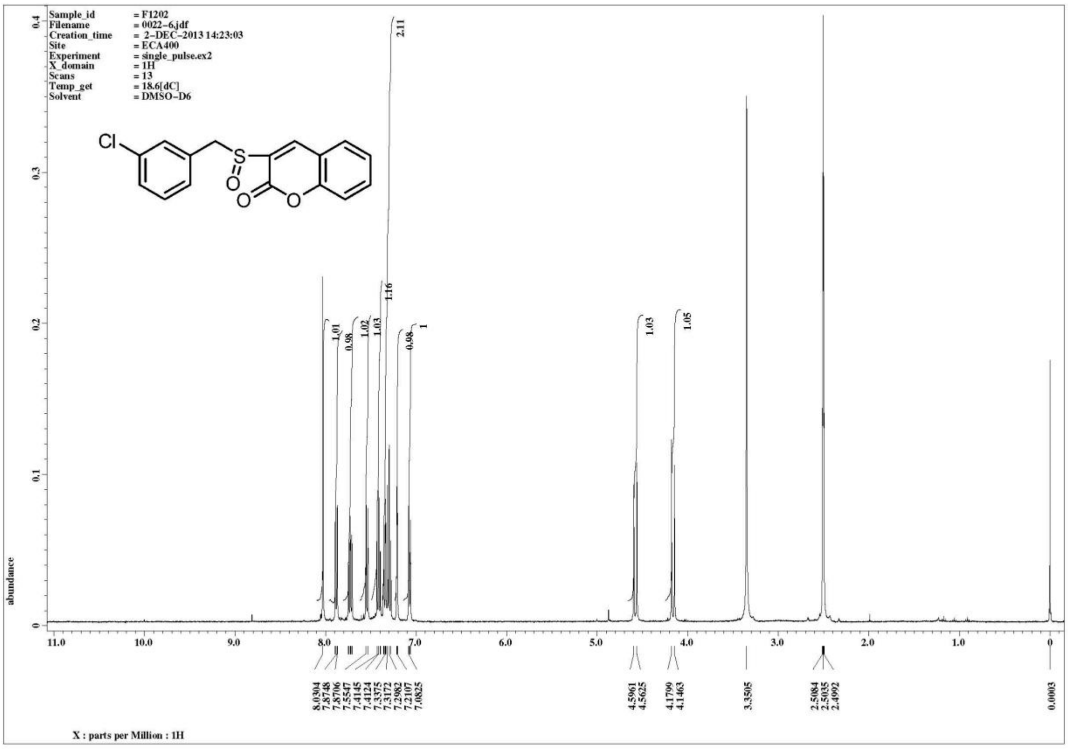

^{13}C-NMR of Compound **5c**:

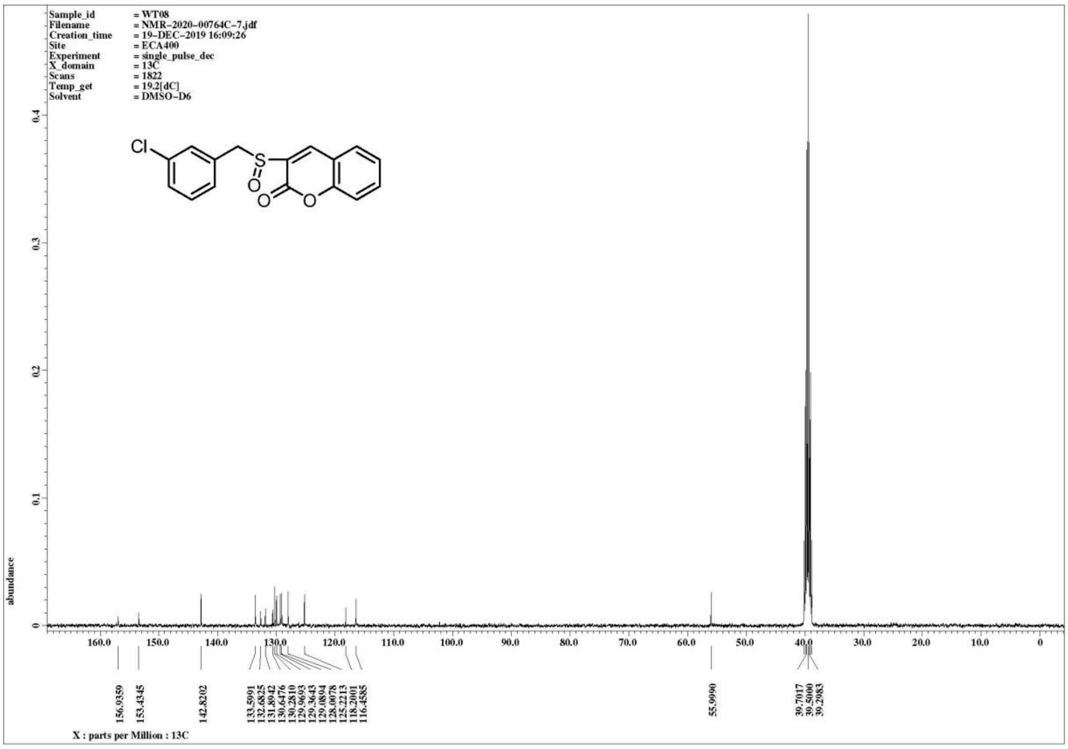

HRMS of Compound **5c**:

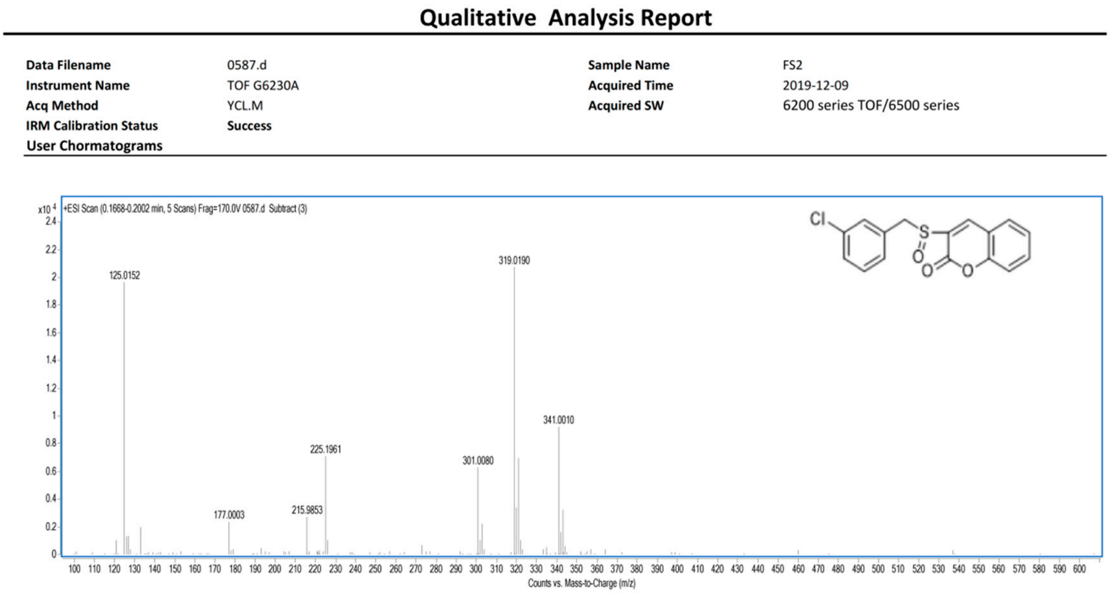

^{1}H-NMR of Compound **5d**:

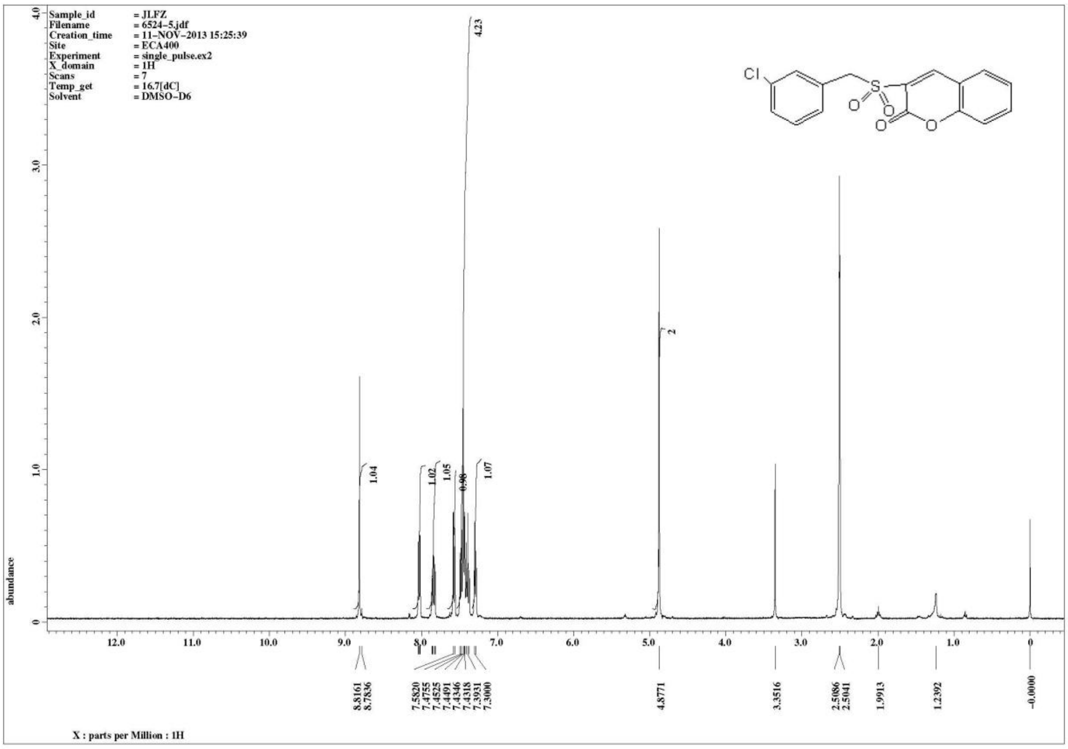

^{1}H-NMR of Compound **5e**:

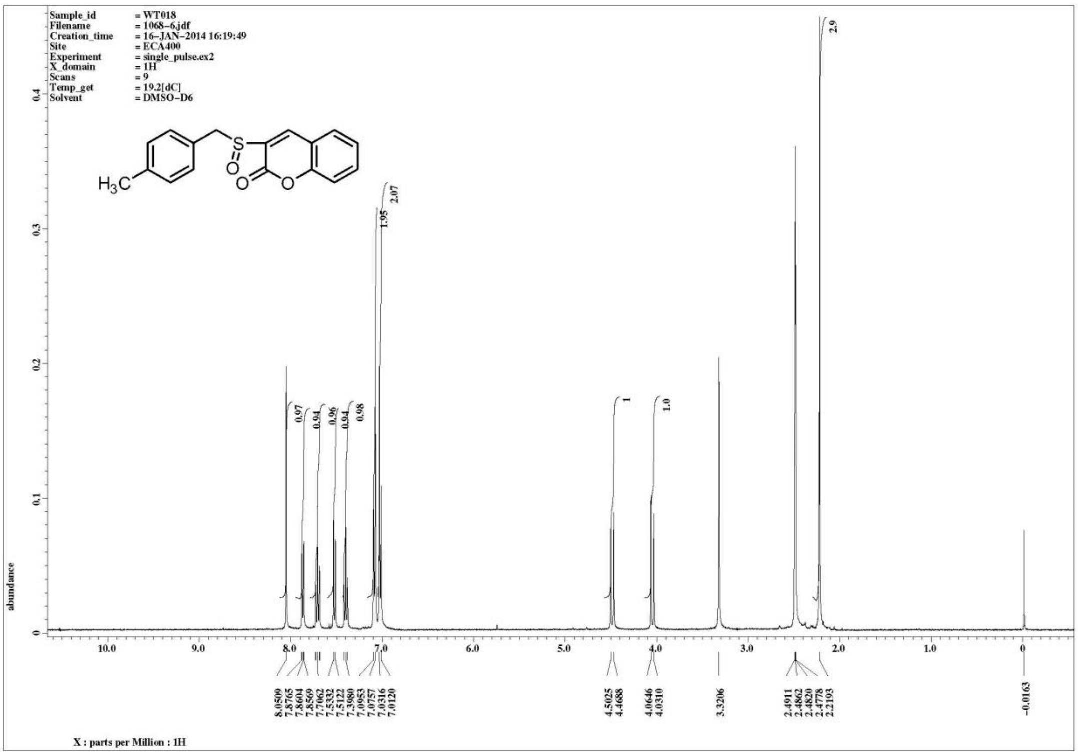

^{13}C-NMR of Compound **5e**:

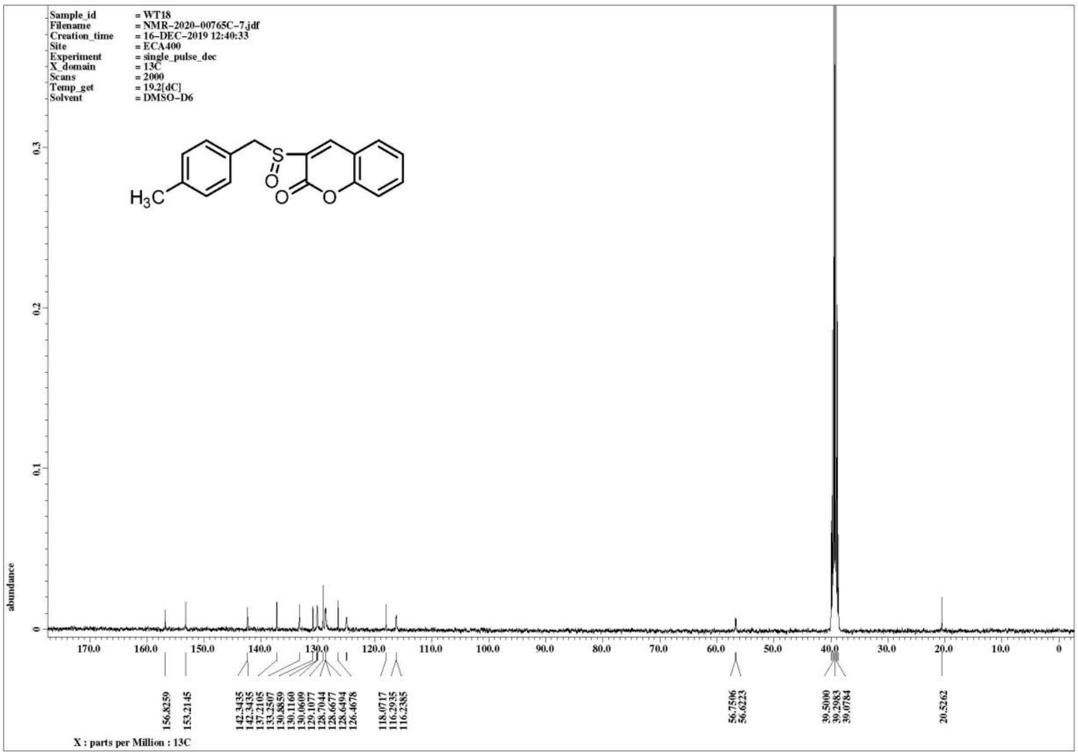

HRMS of Compound **5e**:

Qualitative Analysis Report

**Data Filename**	0588.d	**Sample Name**	FS3
**Instrument Name**	TOF G6230A	**Acquired Time**	2019-12-09
**Acq Method**	YCL.M	**Acquired SW**	6200 series TOF/6500 series
**IRM Calibration Status**	**Success**		
**User Chormatograms**			

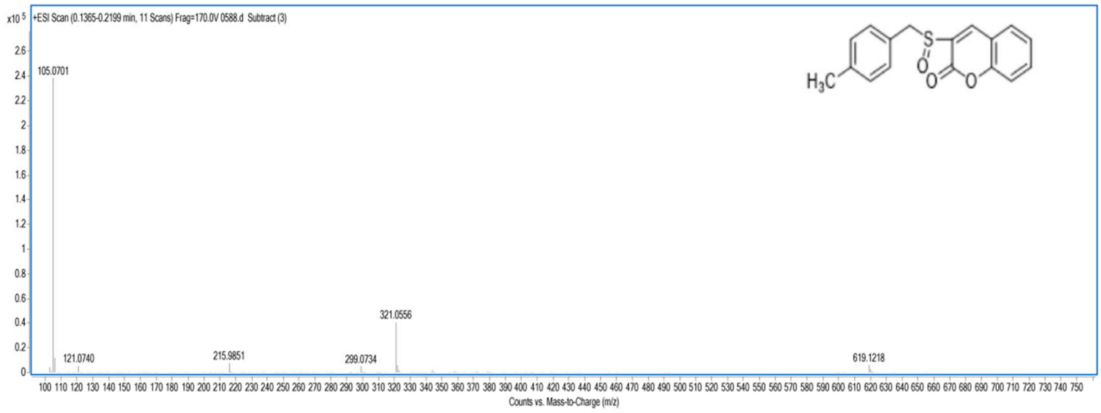

^{1}H-NMR of Compound **5f**:

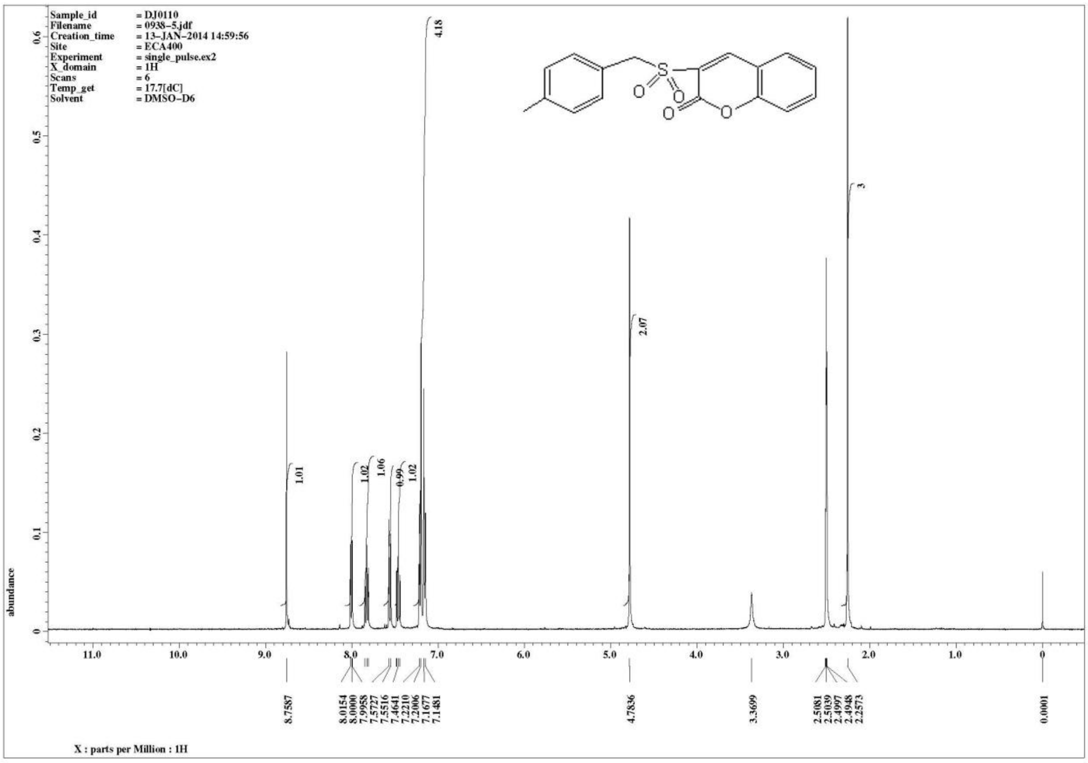

^{1}H-NMR of Compound **5g**:

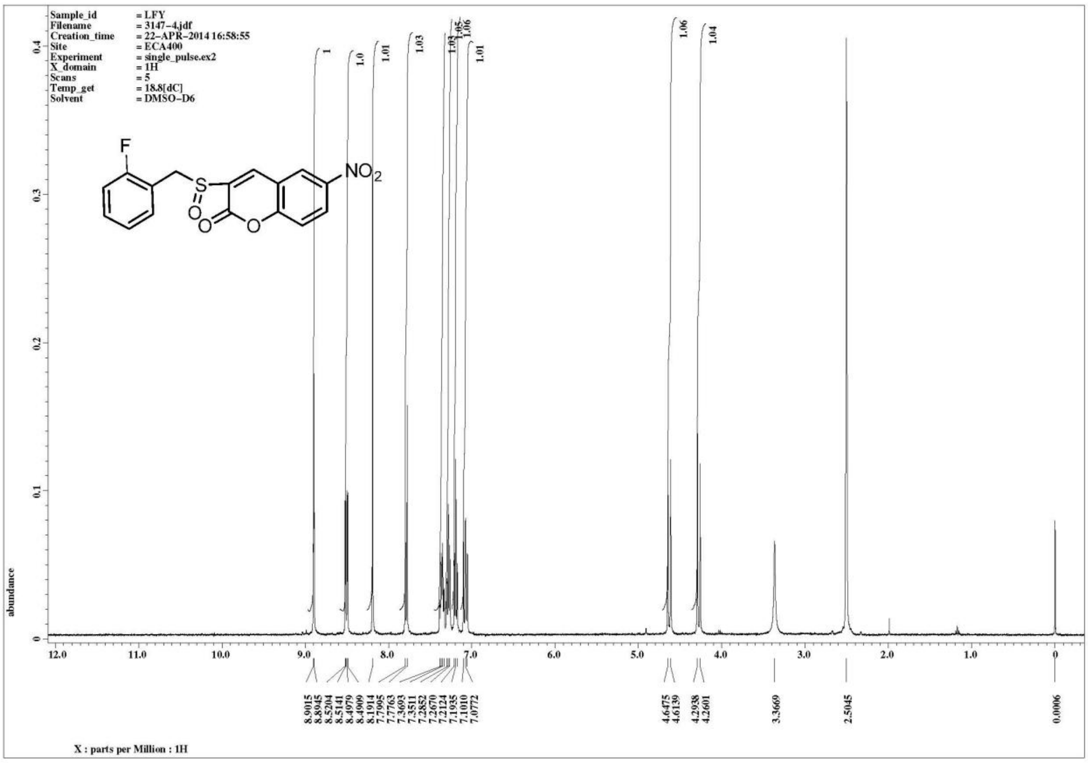

^{13}C-NMR of Compound **5g**:

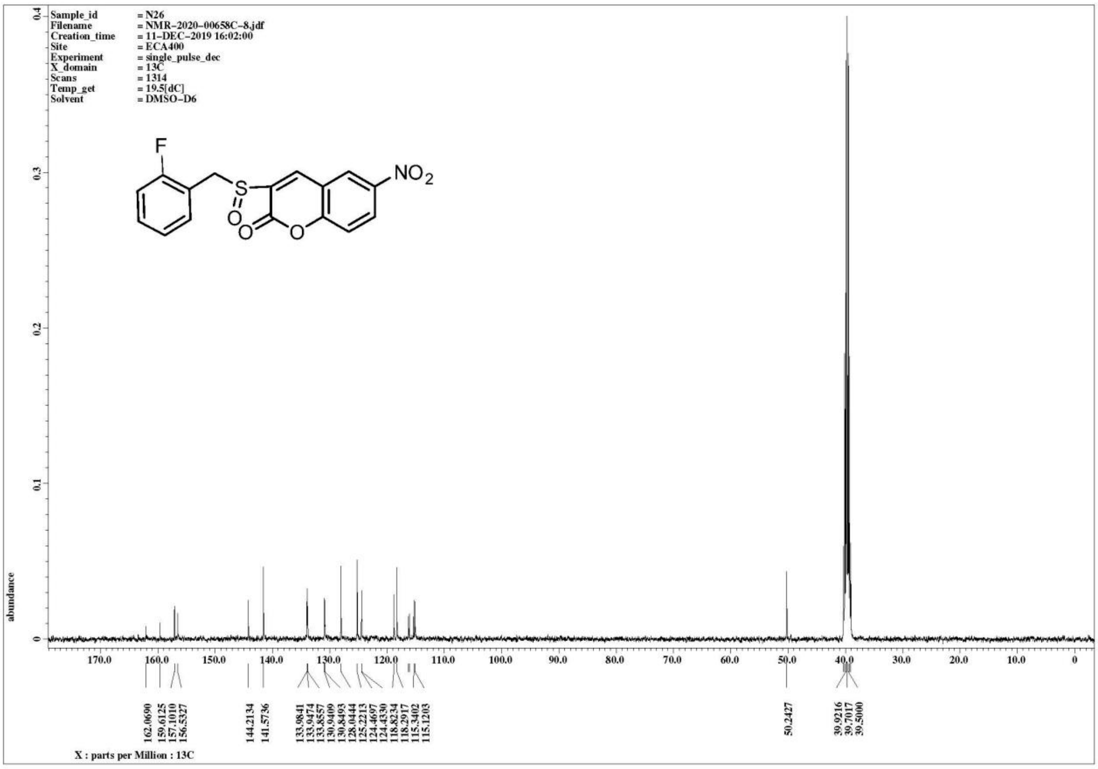

HRMS of Compound **5g**:

Qualitative Analysis Report

**Data Filename**	0592.d	**Sample Name**	FS7
**Instrument Name**	TOF G6230A	**Acquired Time**	2019-12-09
**Acq Method**	YCL.M	**Acquired SW**	6200 series TOF/6500 series
**IRM Calibration Status**	**Success**		
**User Chormatograms**			

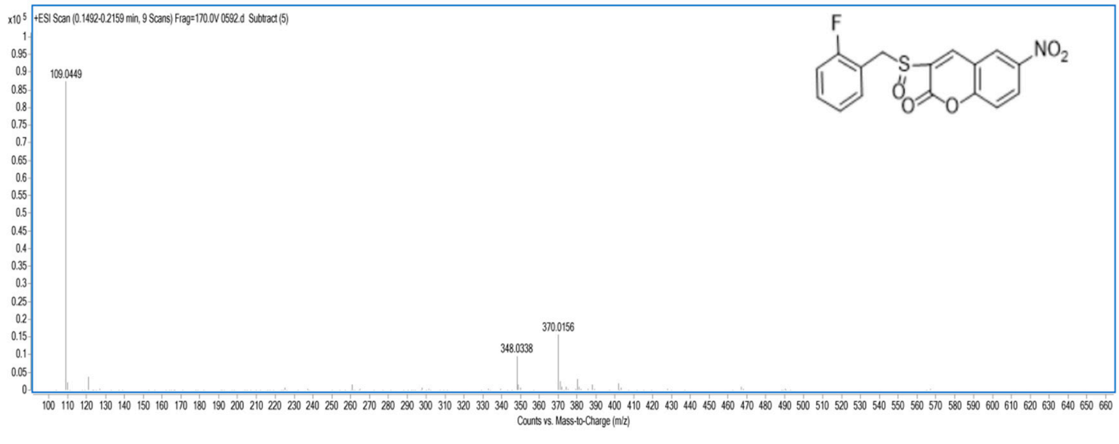

^{1}H-NMR of Compound **5h**:

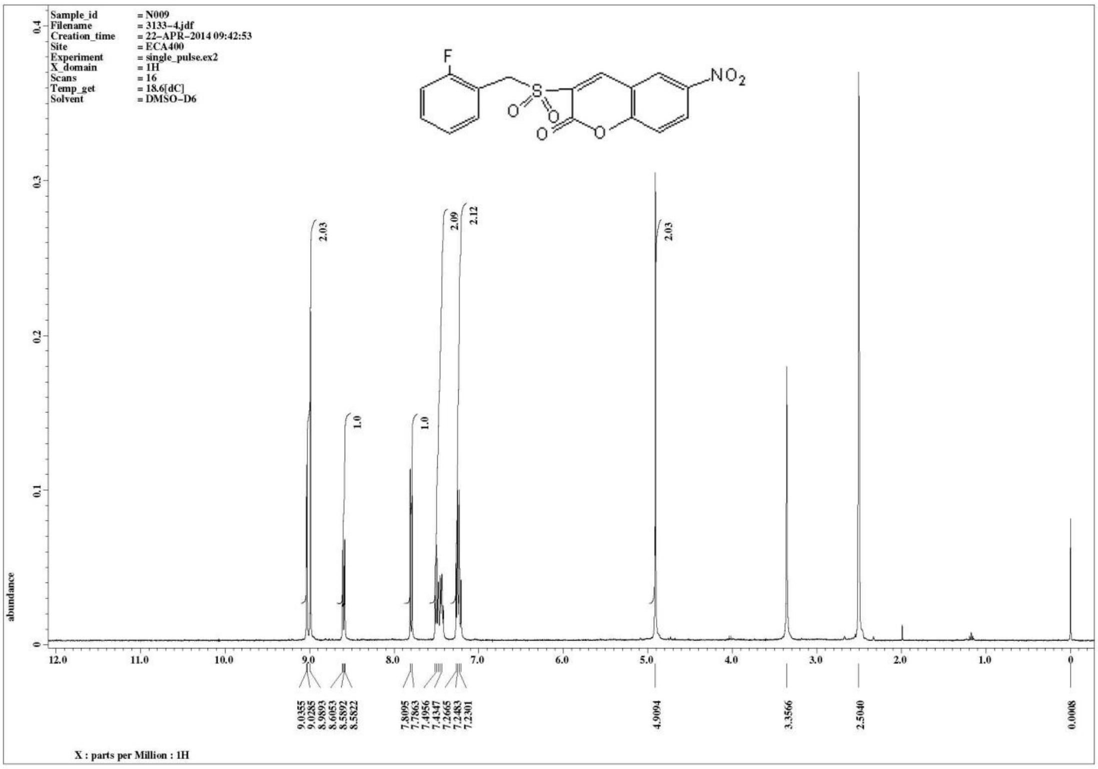

^{1}H-NMR of Compound **5i**:

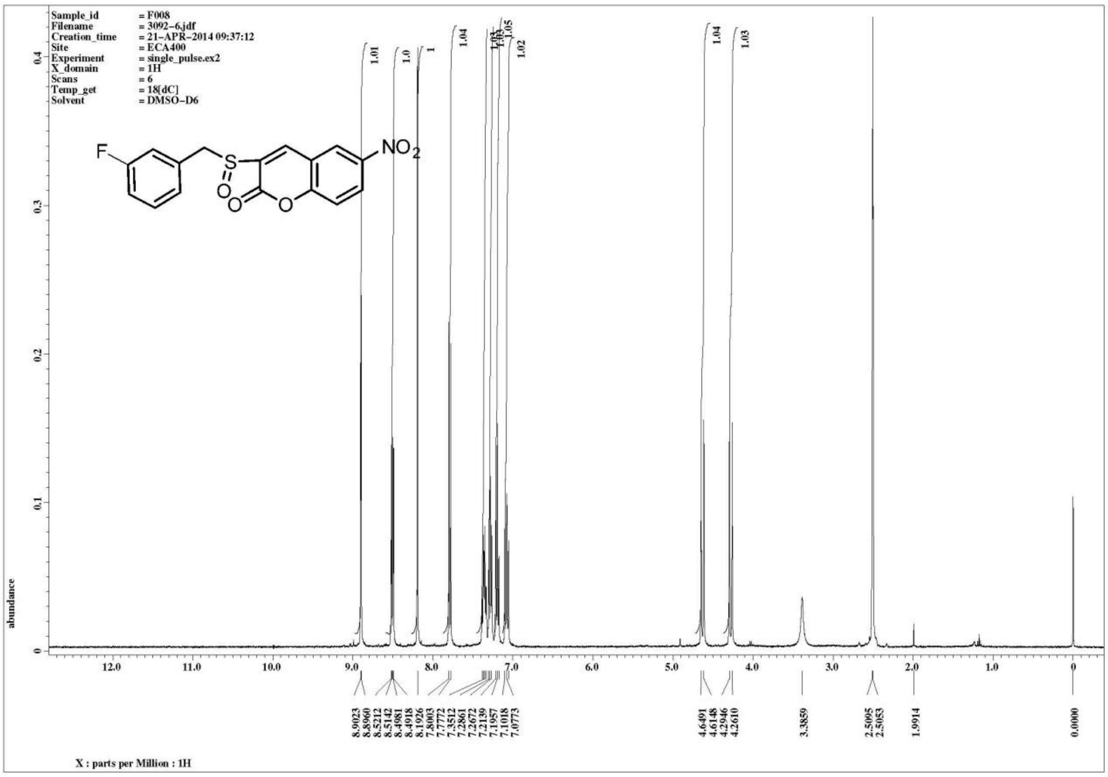

^{13}C-NMR of Compound **5i**:

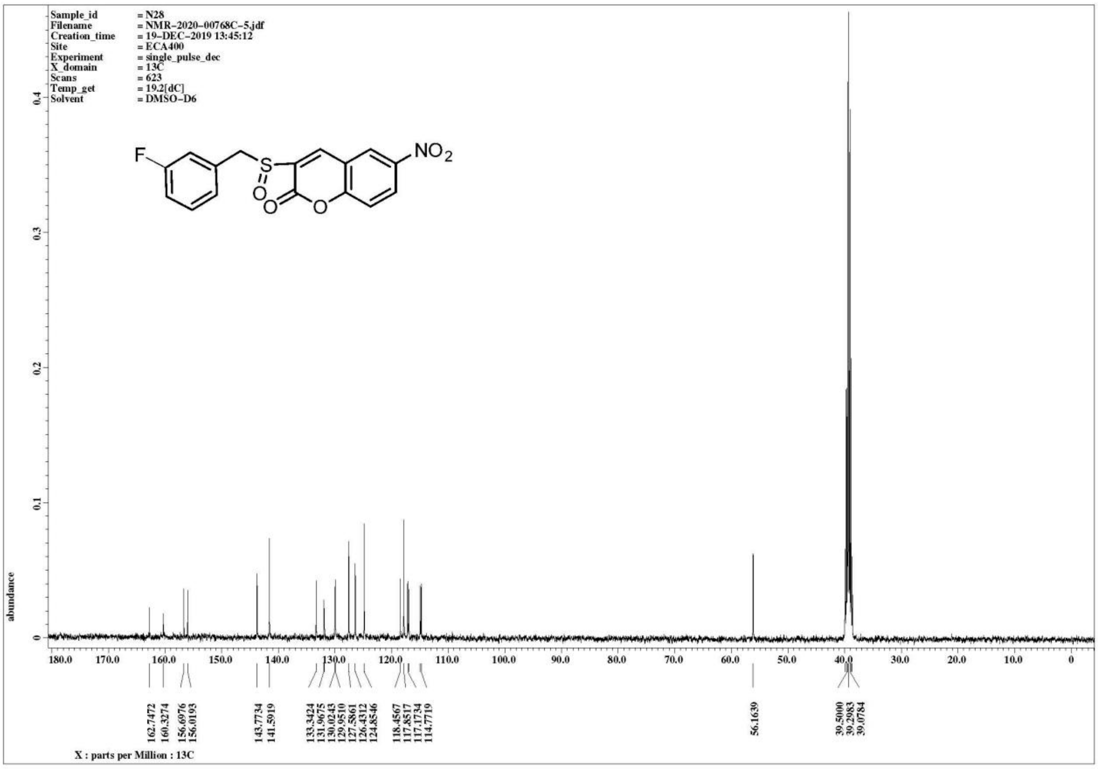

HRMS of Compound **5i**:

Qualitative Analysis Report

**Data Filename**	0593.d	**Sample Name**	FS8
**Instrument Name**	TOF G6230A	**Acquired Time**	2019-12-09
**Acq Method**	YCL.M	**Acquired SW**	6200 series TOF/6500 series
**IRM Calibration Status**	**Success**		
**User Chormatograms**			

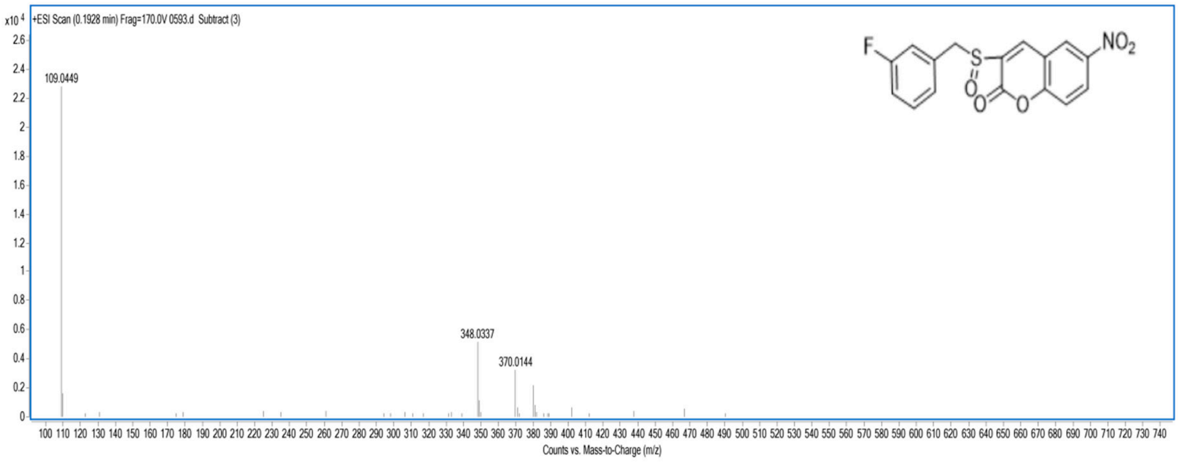

^{1}H-NMR of Compound **5j**:

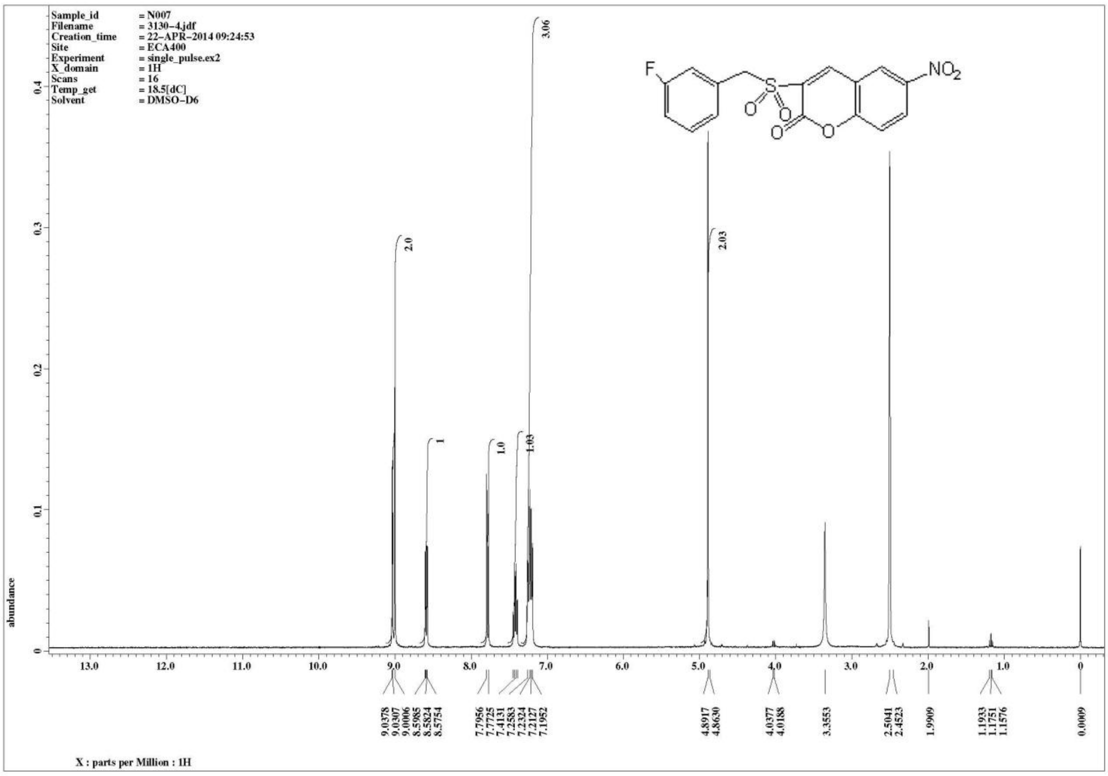

^{1}H-NMR of Compound **5k**:

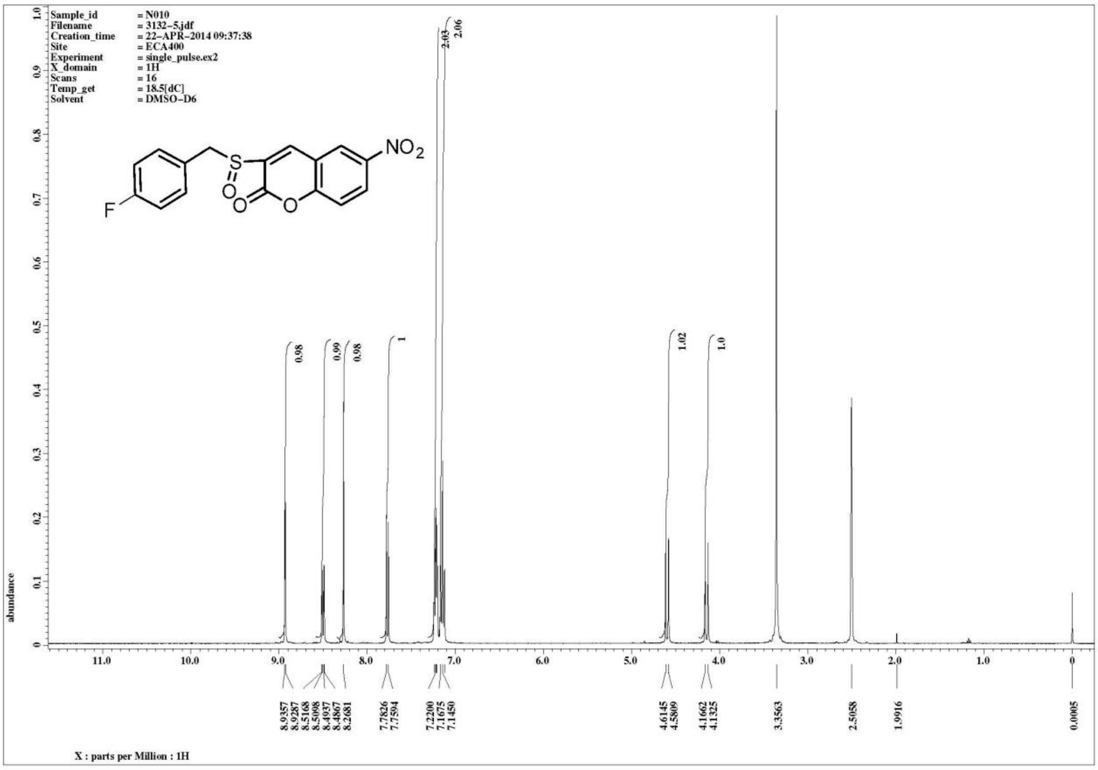

^{13}C-NMR of Compound **5k**:

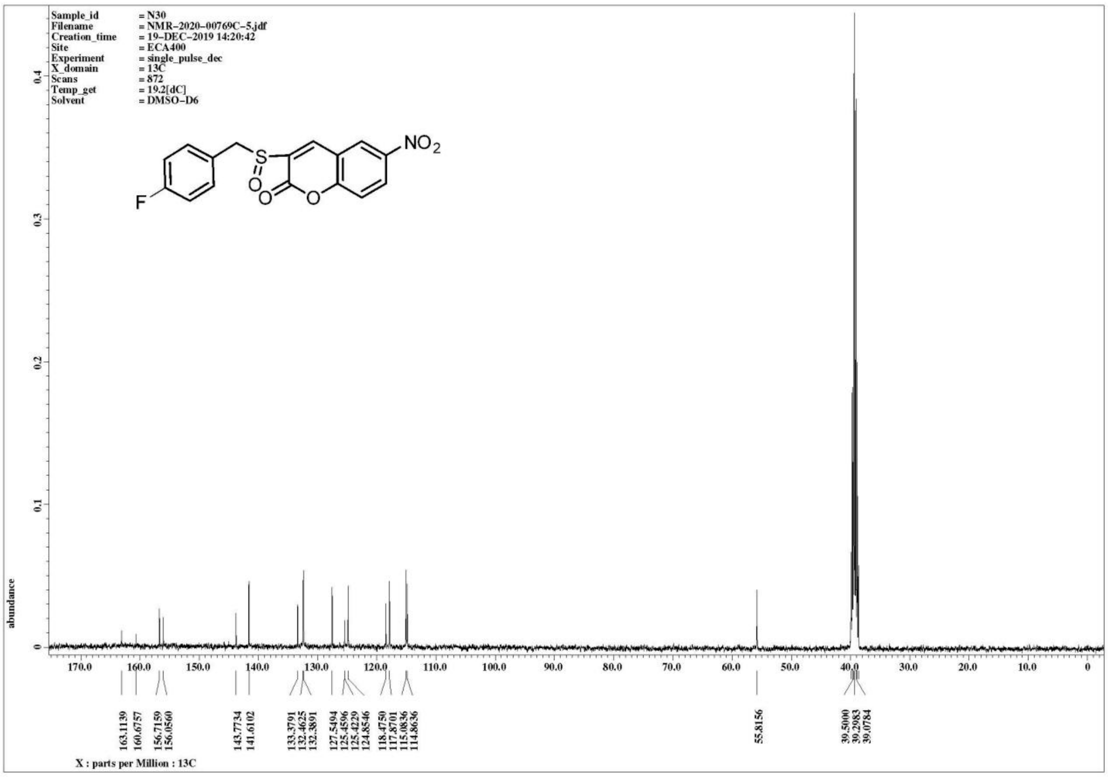

HRMS of Compound **5k**:

Qualitative Analysis Report

**Data Filename**	0594.d	**Sample Name**	FS9
**Instrument Name**	TOF G6230A	**Acquired Time**	2019-12-09
**Acq Method**	YCL.M	**Acquired SW**	6200 series TOF/6500 series
**IRM Calibration Status**	**Success**		
**User Chormatograms**			

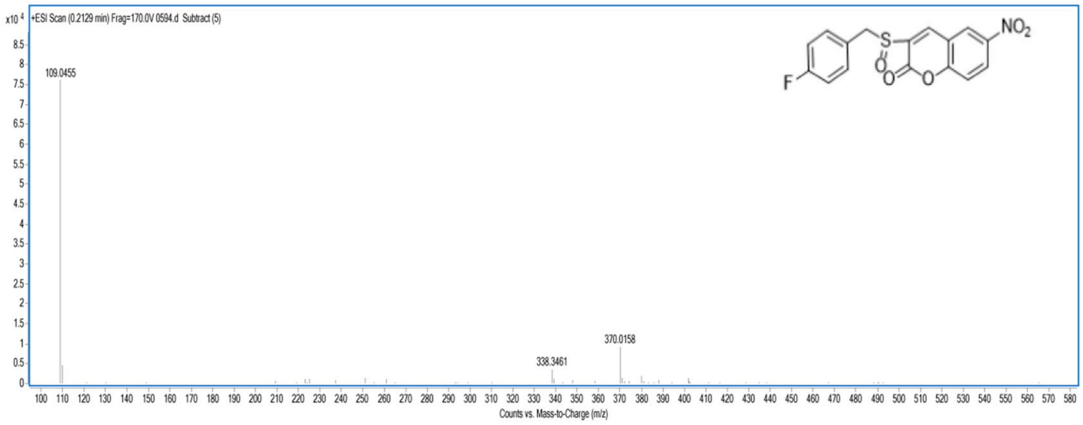

^{1}H-NMR of Compound **5l**:

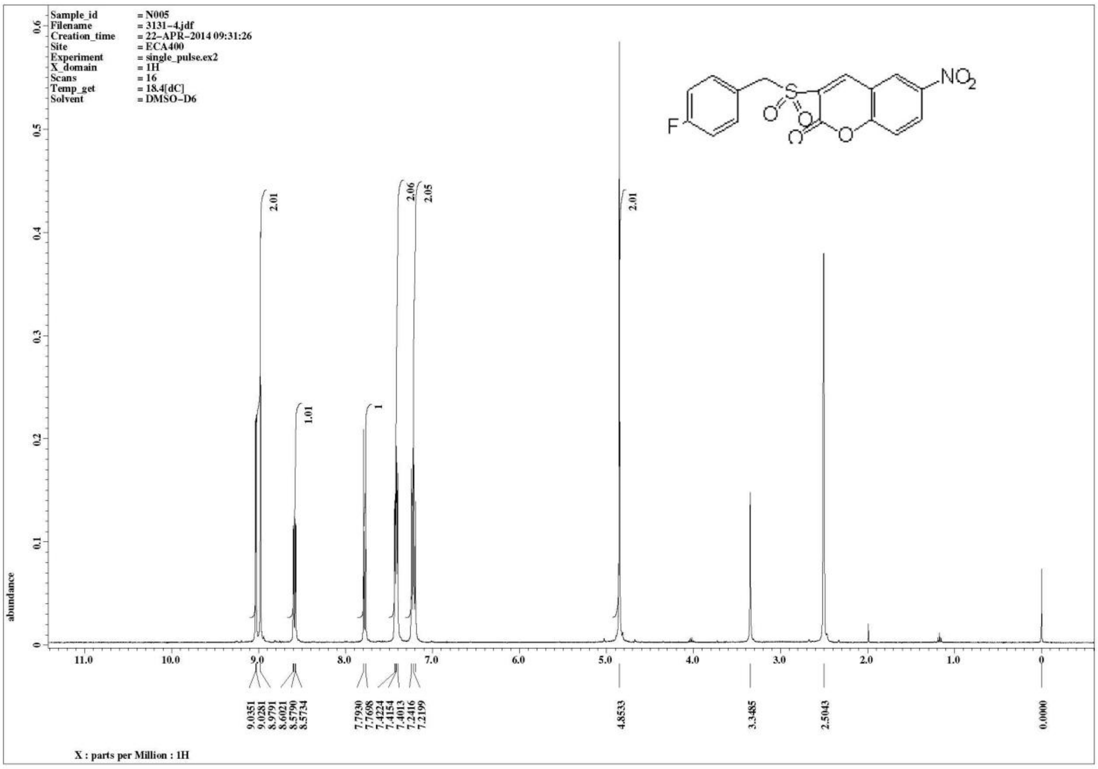

^{1}H-NMR of Compound **5m**:

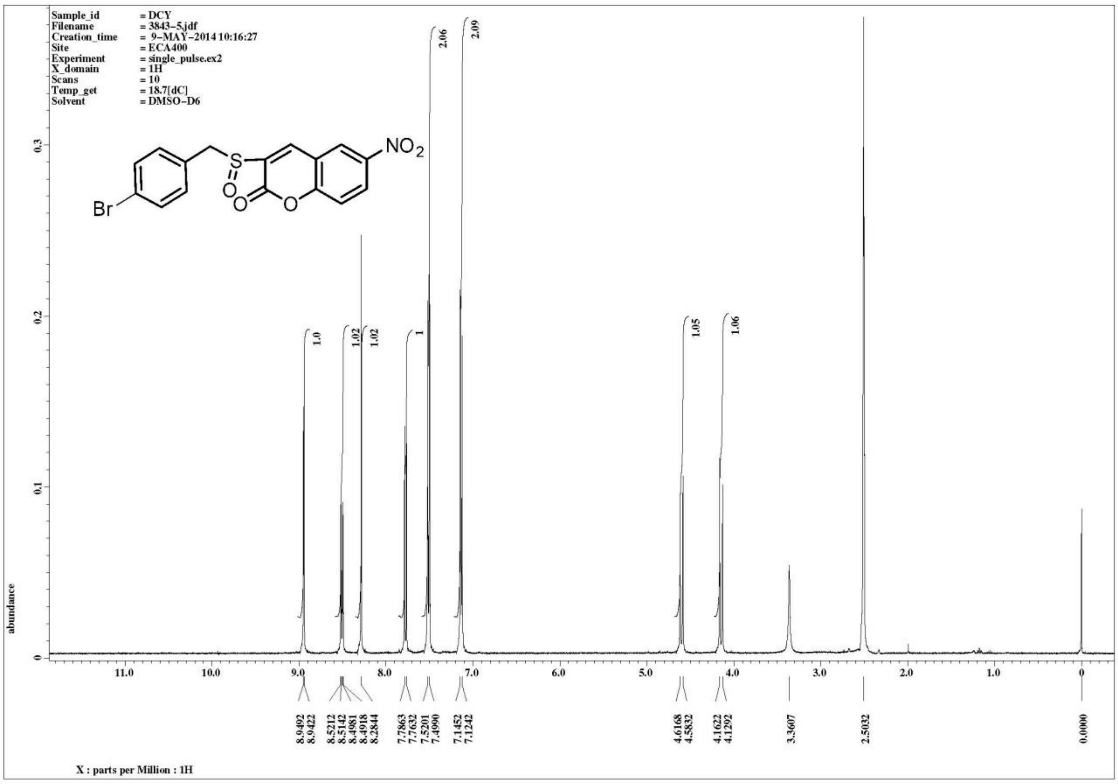

^{13}C-NMR of Compound **5m**:

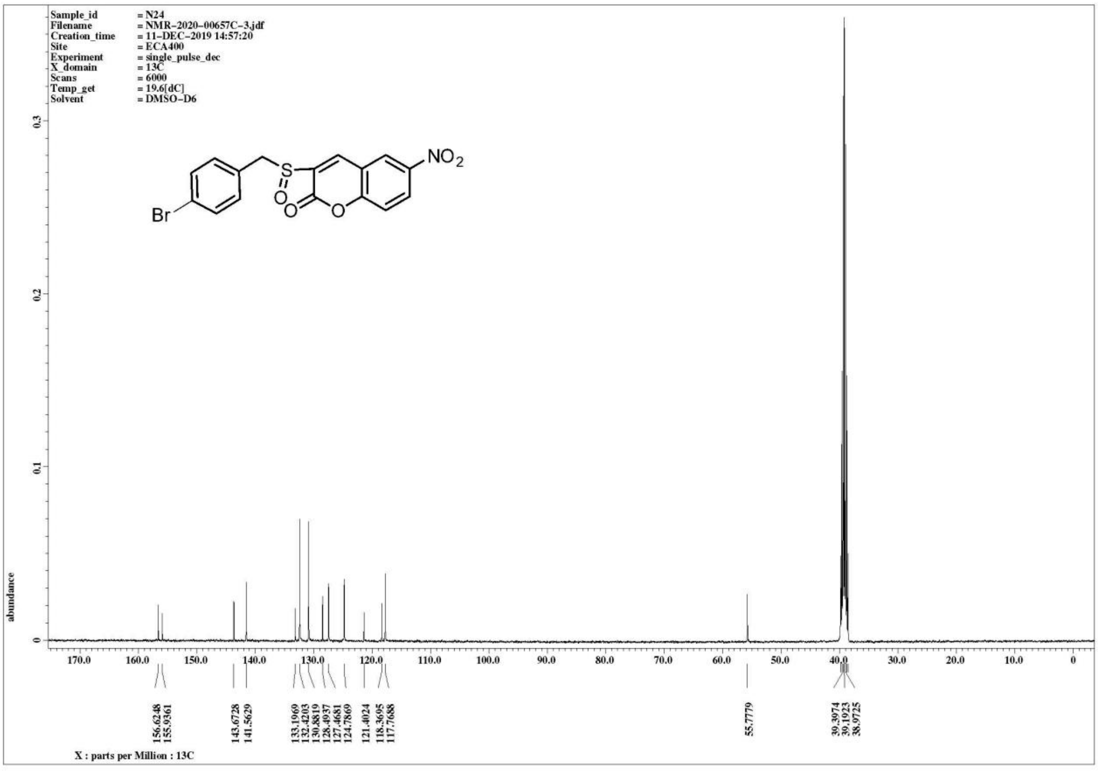

HRMS of Compound **5m**:

Qualitative Analysis Report

**Data Filename**	0591.d	**Sample Name**	FS6
**Instrument Name**	TOF G6230A	**Acquired Time**	2019-12-09
**Acq Method**	YCL.M	**Acquired SW**	6200 series TOF/6500 series
**IRM Calibration Status**	**Success**		
**User Chormatograms**			

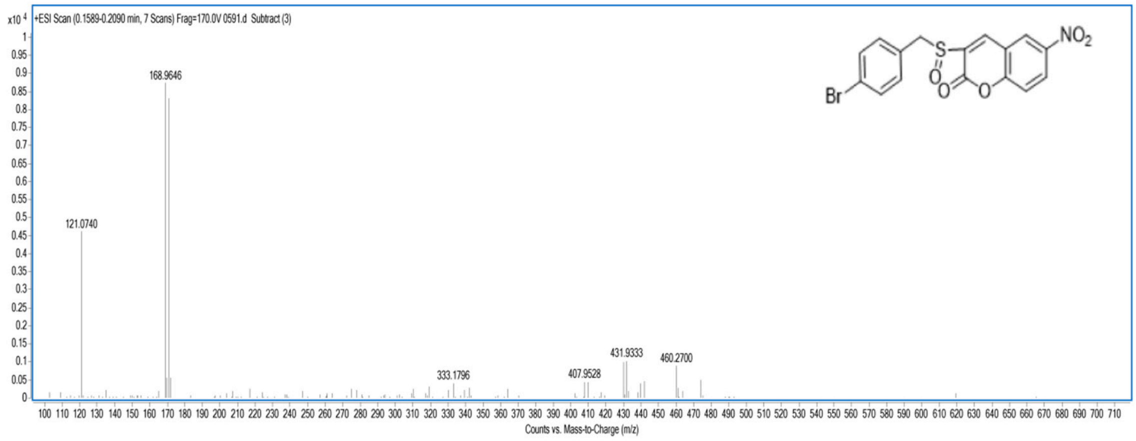

^{1}H-NMR of Compound **5n**:

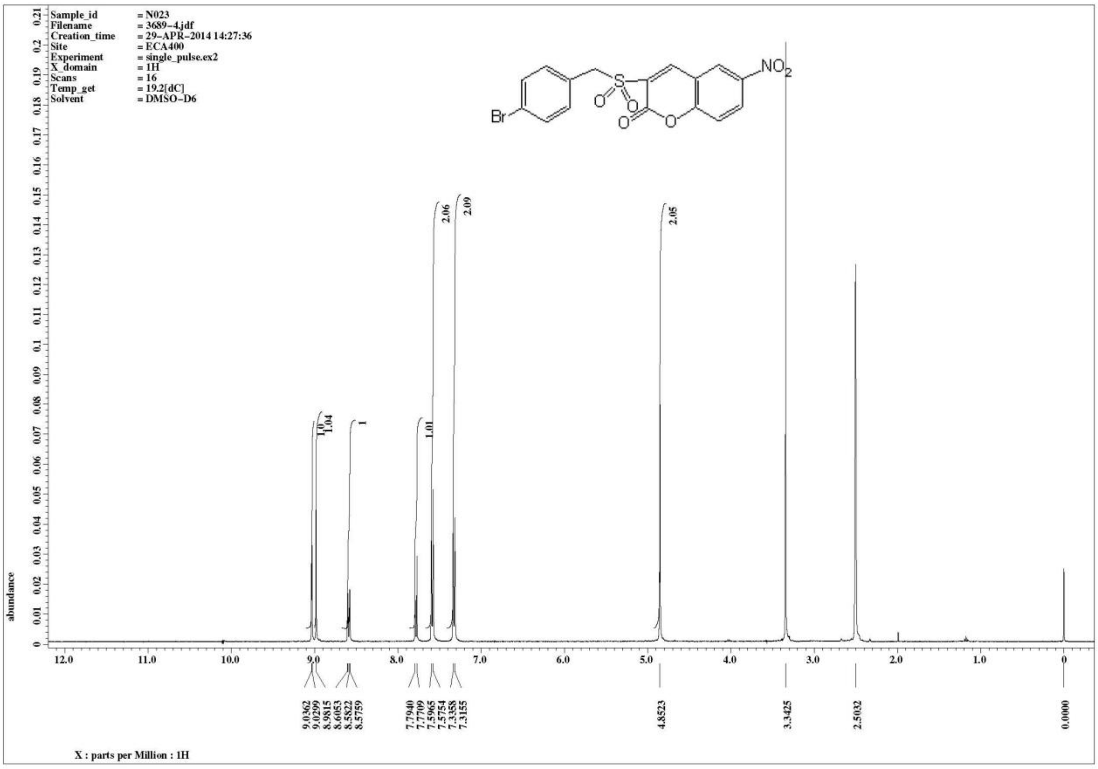

^{13}C-NMR of Compound **5n**:

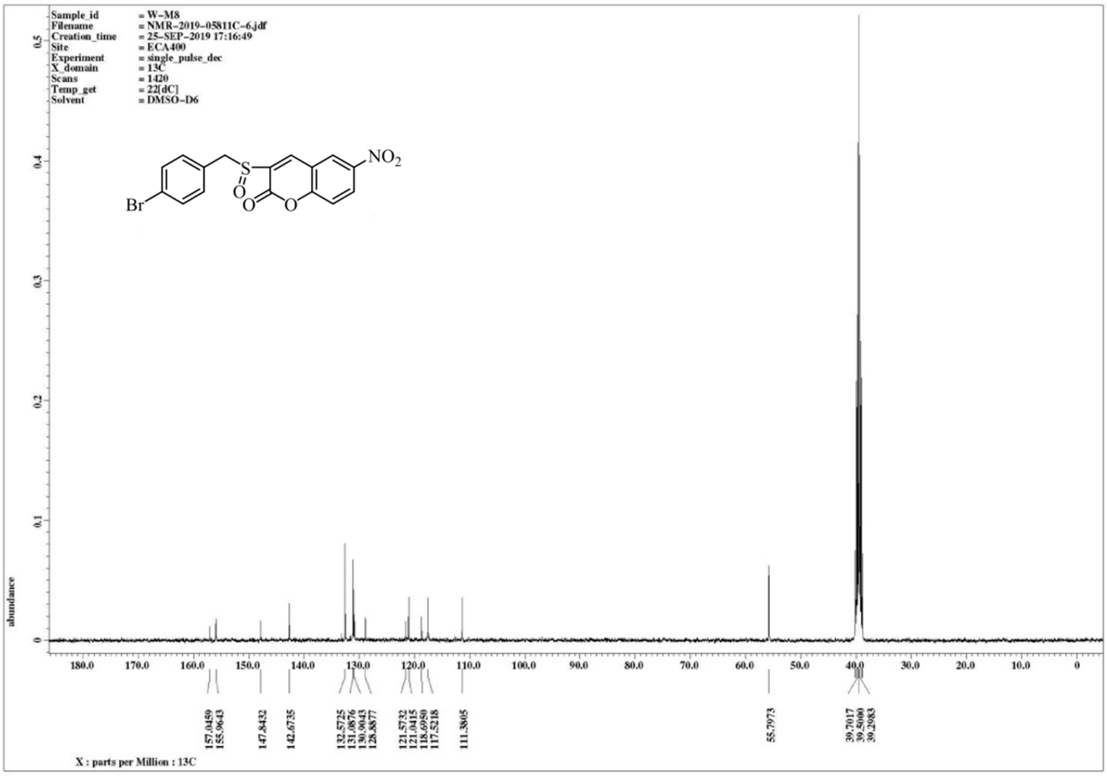

^{1}H-NMR of Compound **5o**:

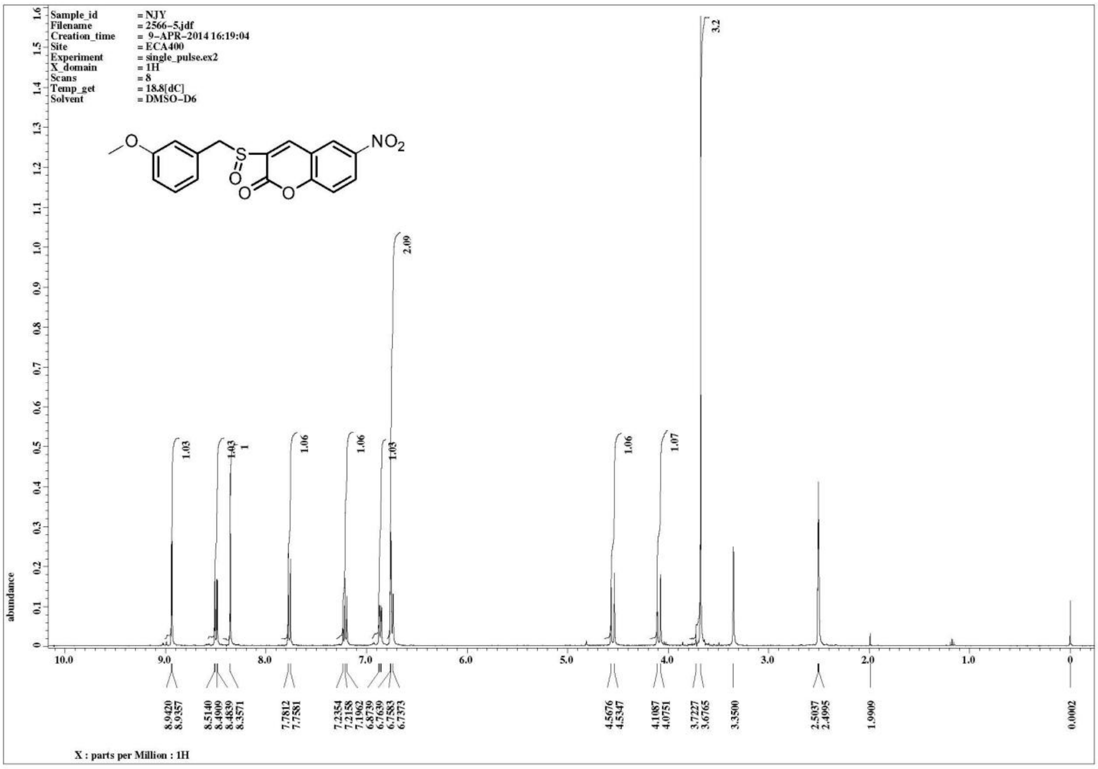

^{13}C-NMR of Compound **5o**:

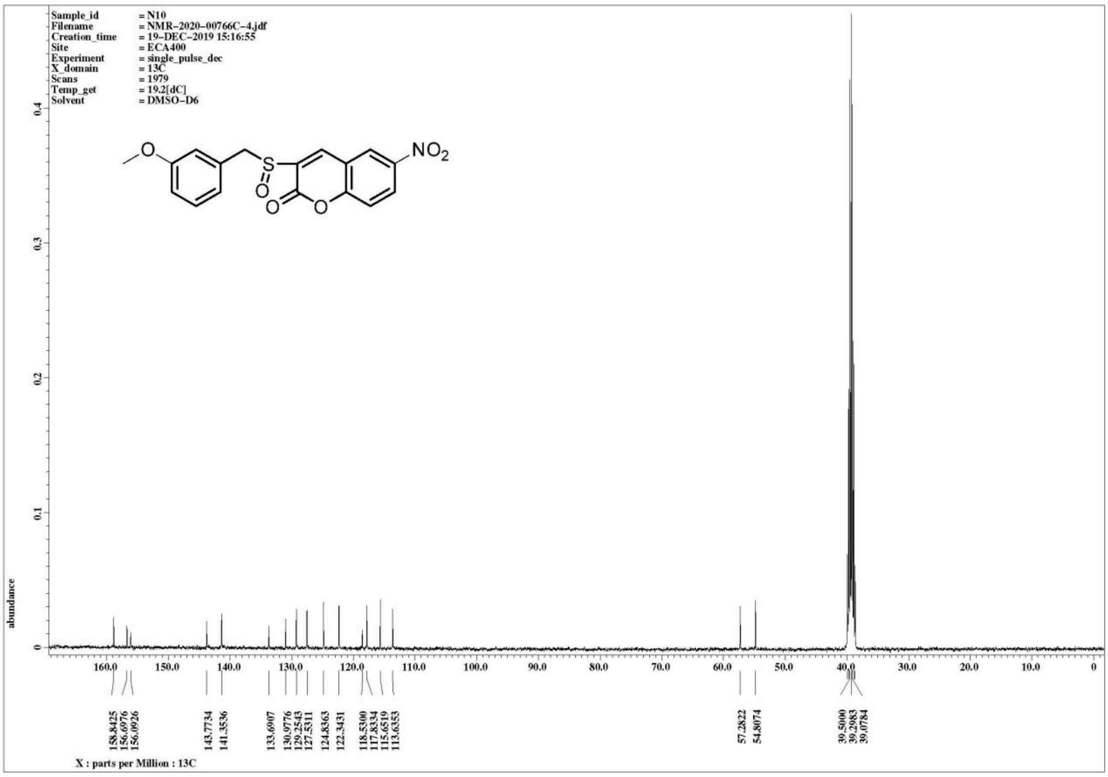

HRMS of Compound **5o**:

Qualitative Analysis Report

**Data Filename**	0589.d	**Sample Name**	FS4
**Instrument Name**	TOF G6230A	**Acquired Time**	2019-12-09
**Acq Method**	YCL.M	**Acquired SW**	6200 series TOF/6500 series
**IRM Calibration Status**	**Success**		
**User Chromatograms**			

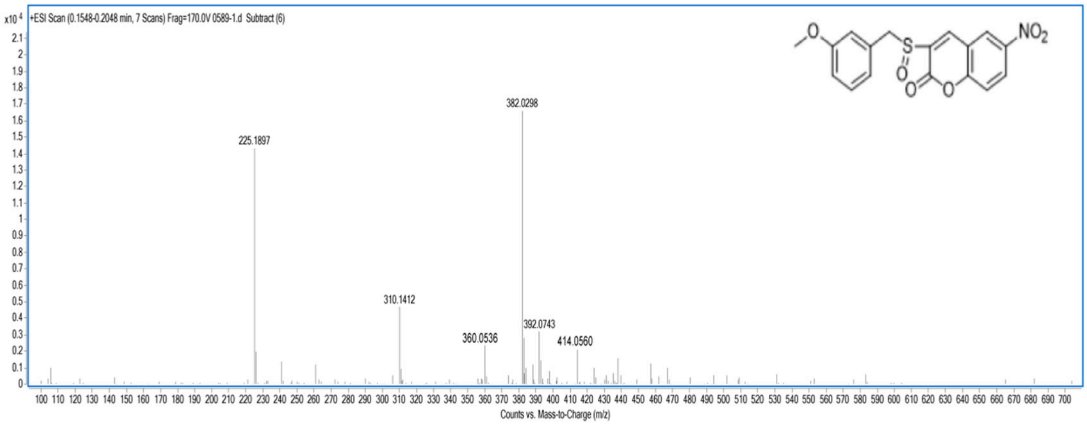

^{1}H-NMR of Compound **5p**:

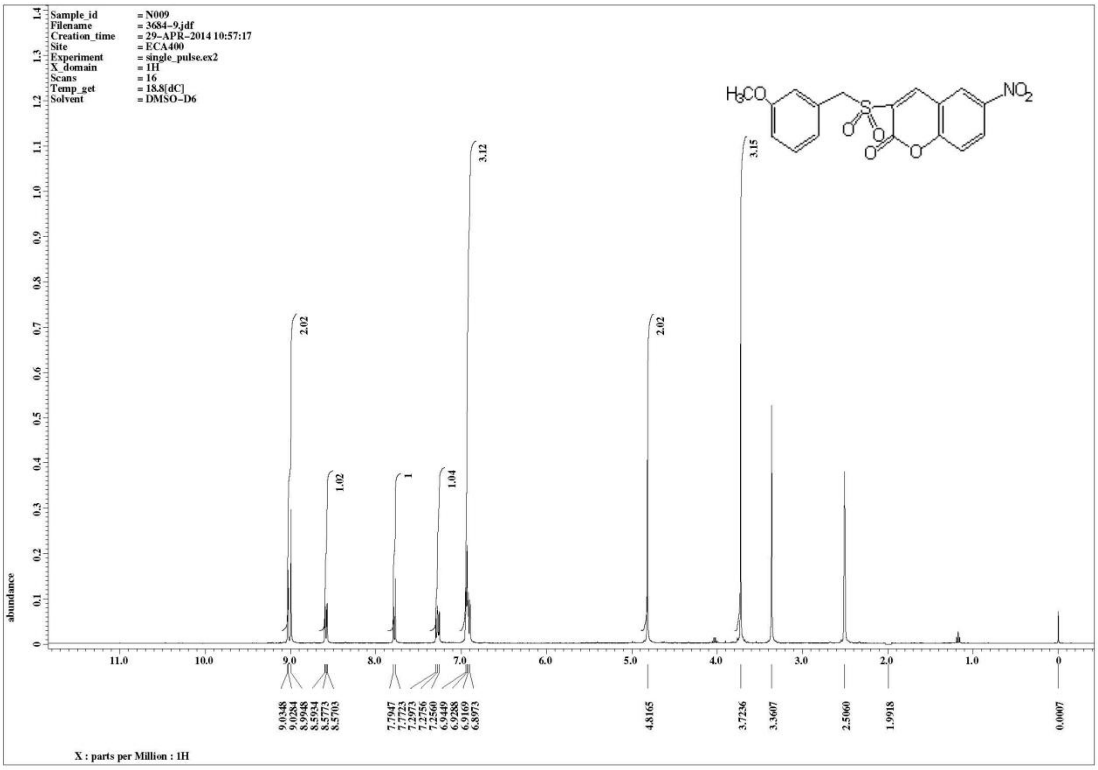

^{1}H-NMR of Compound **5q**:

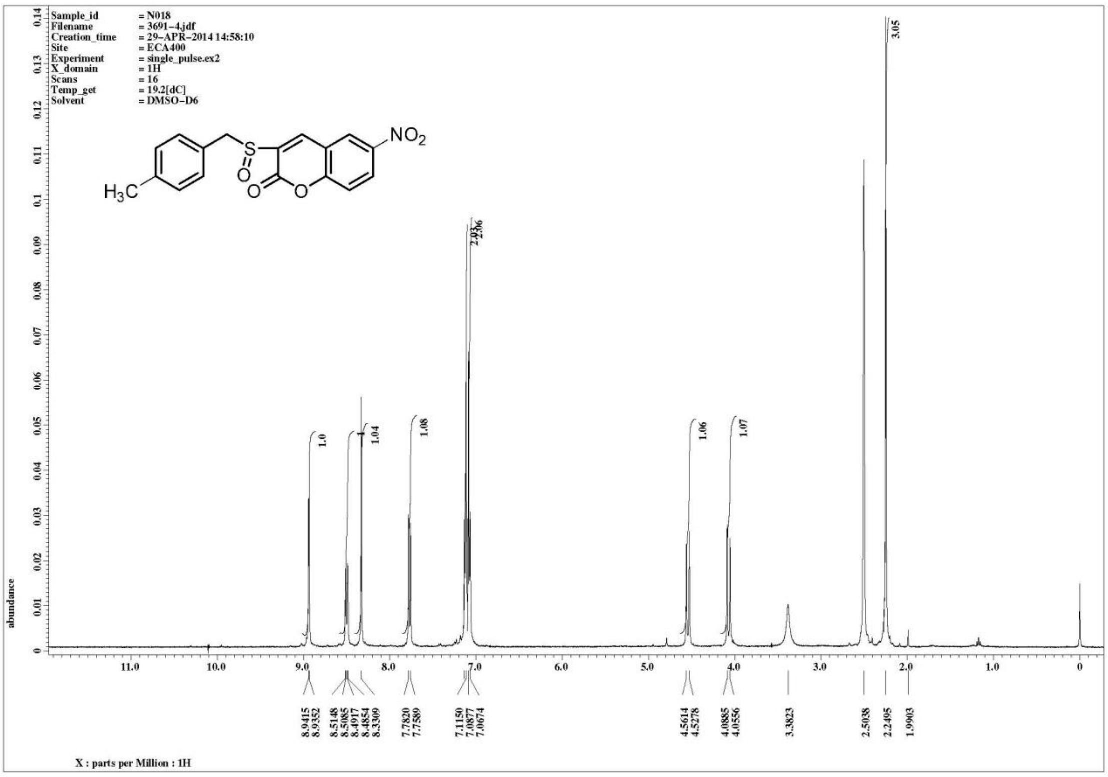

^{13}C-NMR of Compound **5q**:

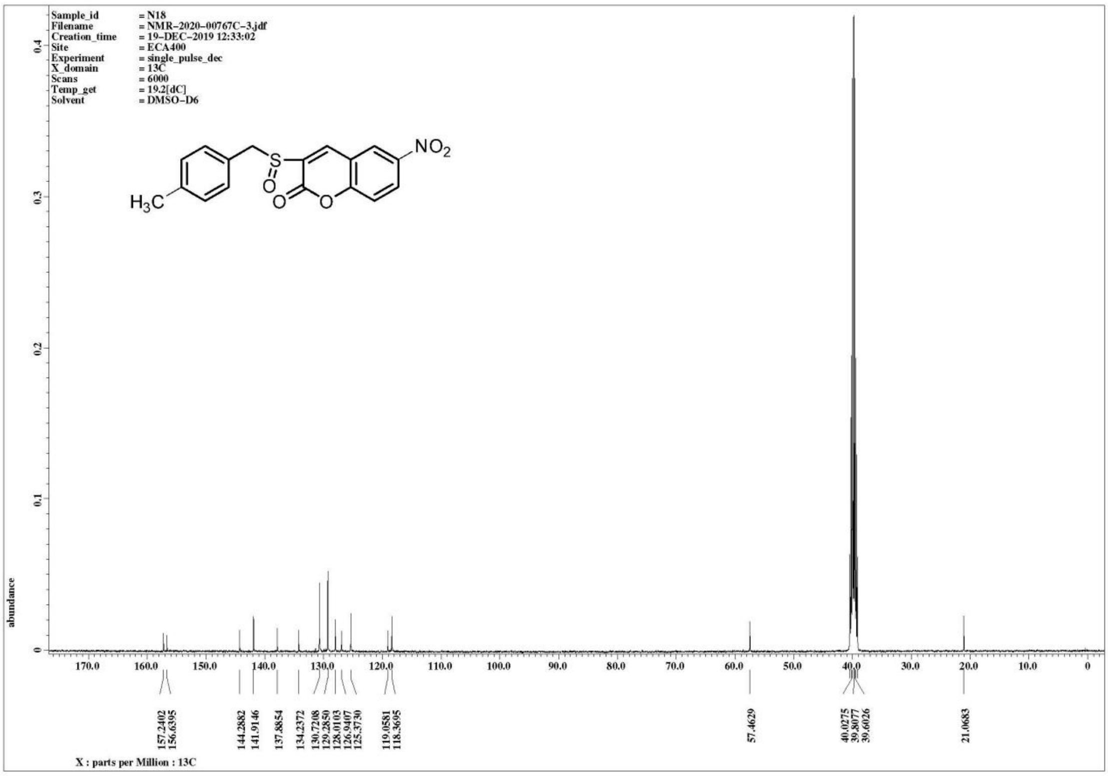

HRMS of Compound **5q**:

Qualitative Analysis Report

**Data Filename**	0590.d	**Sample Name**	FS5
**Instrument Name**	TOF G6230A	**Acquired Time**	2019-12-09
**Acq Method**	YCL.M	**Acquired SW**	6200 series TOF/6500 series
**IRM Calibration Status**	**Success**		
**User Chormatograms**			

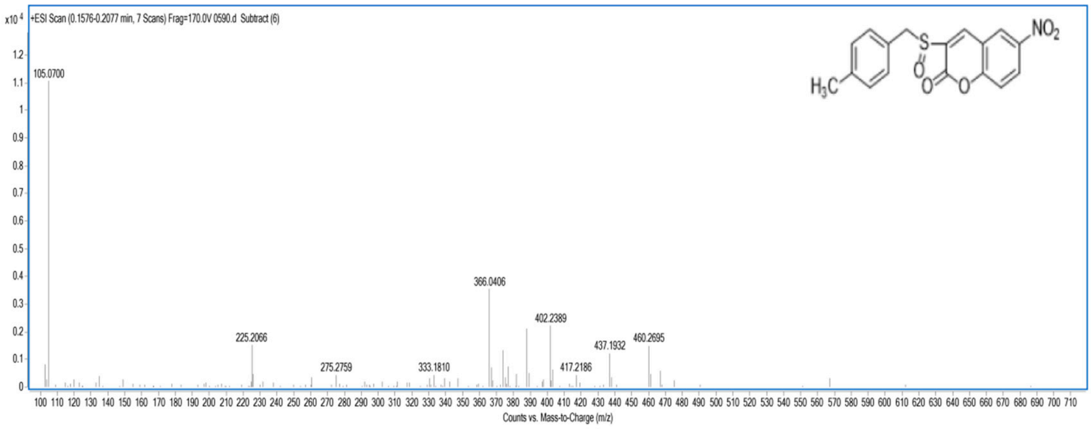

^{1}H-NMR of Compound **5r**:

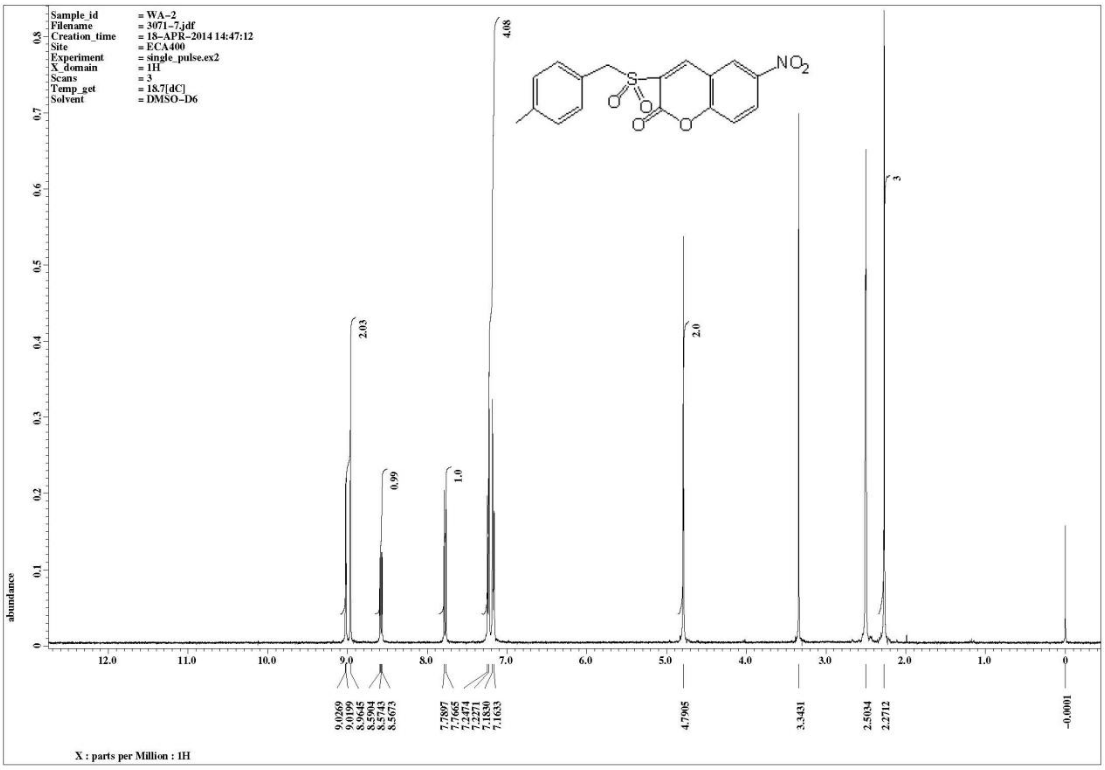

^{1}H-NMR of Compound **5s**:

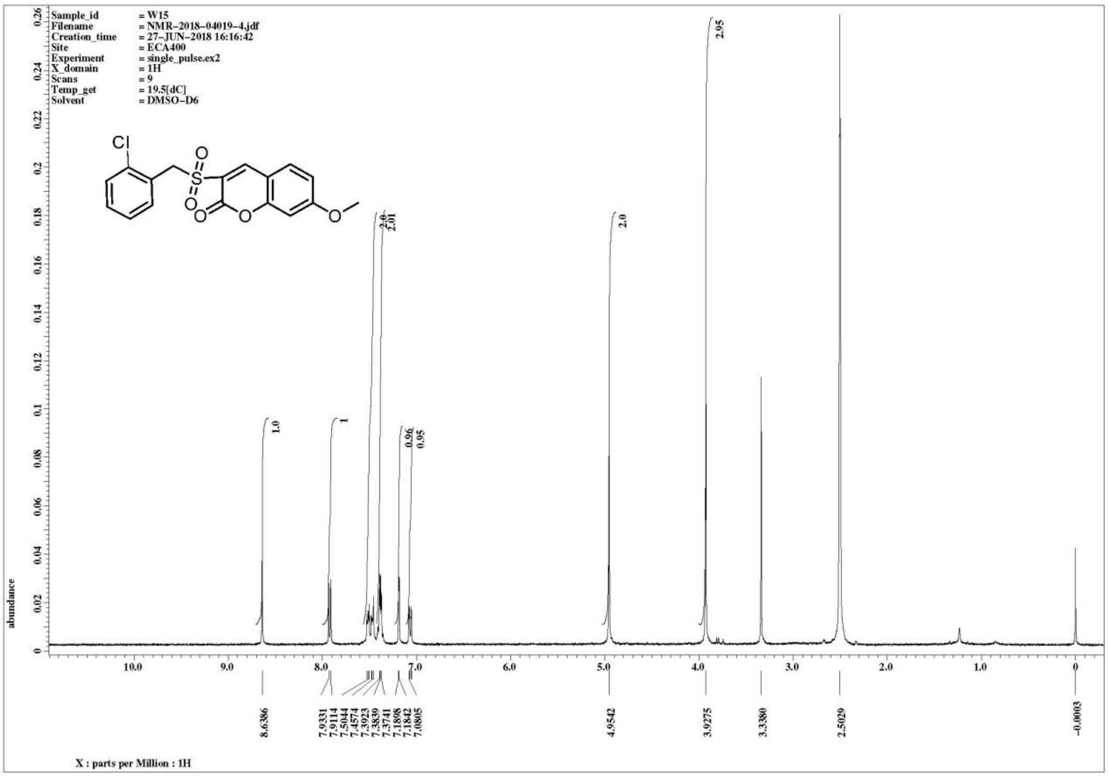

^{13}C-NMR of Compound **5s**:

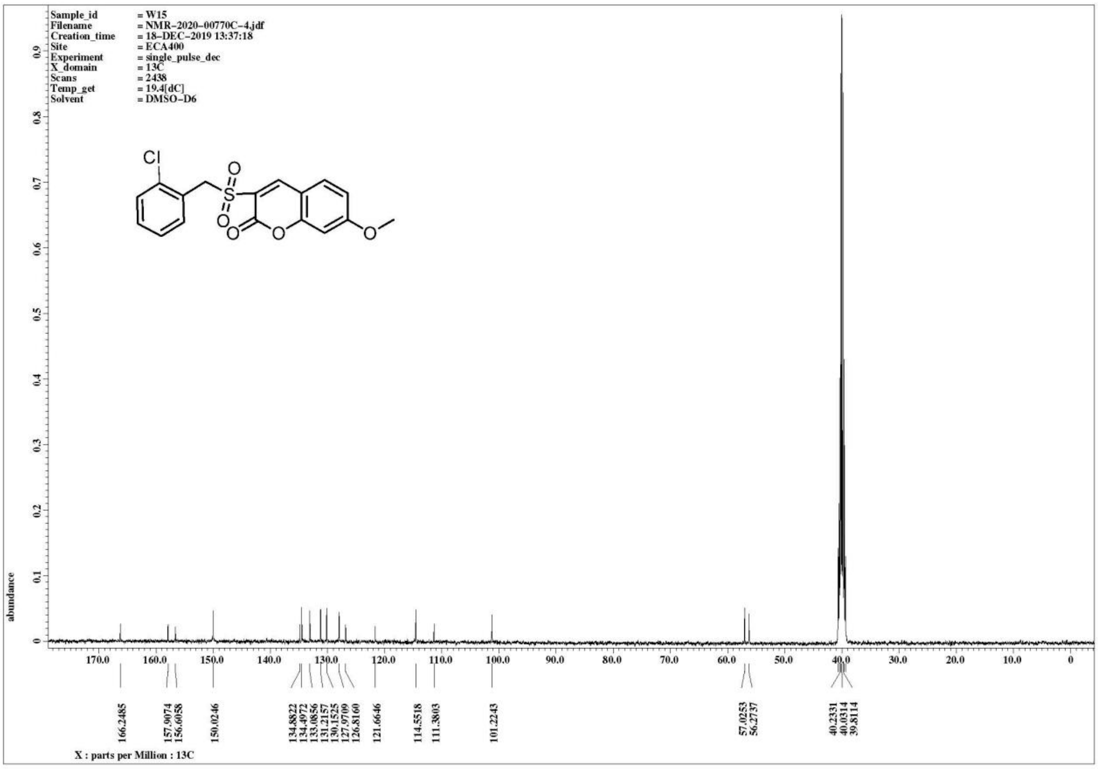

HRMS of Compound **5s**:

Qualitative Analysis Report

**Data Filename**	0595.d	**Sample Name**	FS10
**Instrument Name**	TOF G6230A	**Acquired Time**	2019-12-09
**Acq Method**	YCL.M	**Acquired SW**	6200 series TOF/6500 series
**IRM Calibration Status**	**Success**		
**User Chormatograms**			

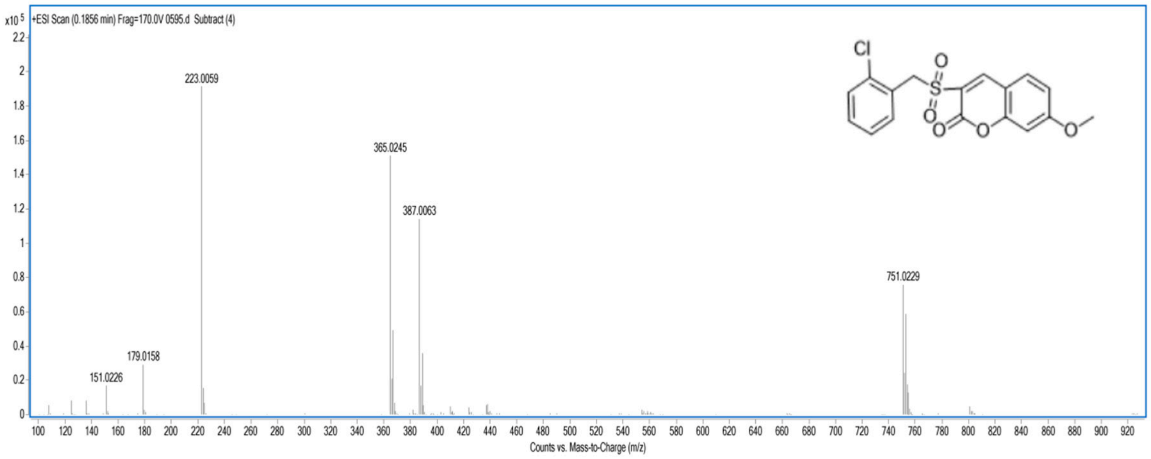

^{1}H-NMR of Compound **5t**:

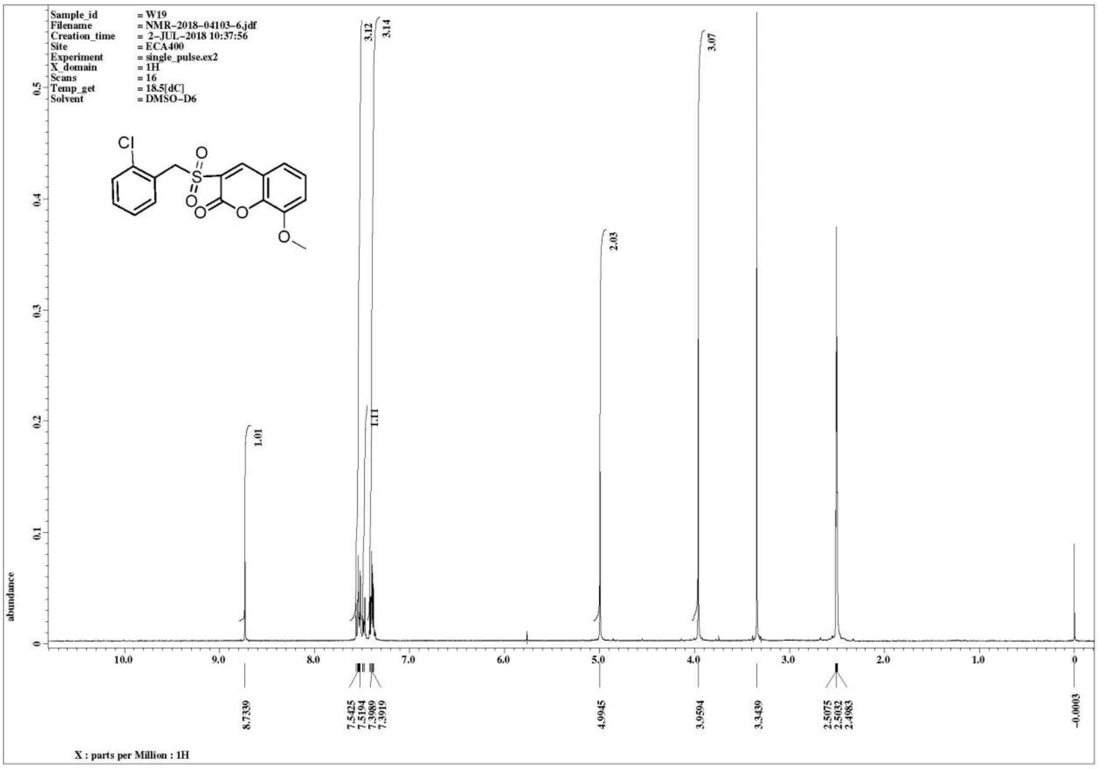

^{13}C-NMR of Compound **5t**:

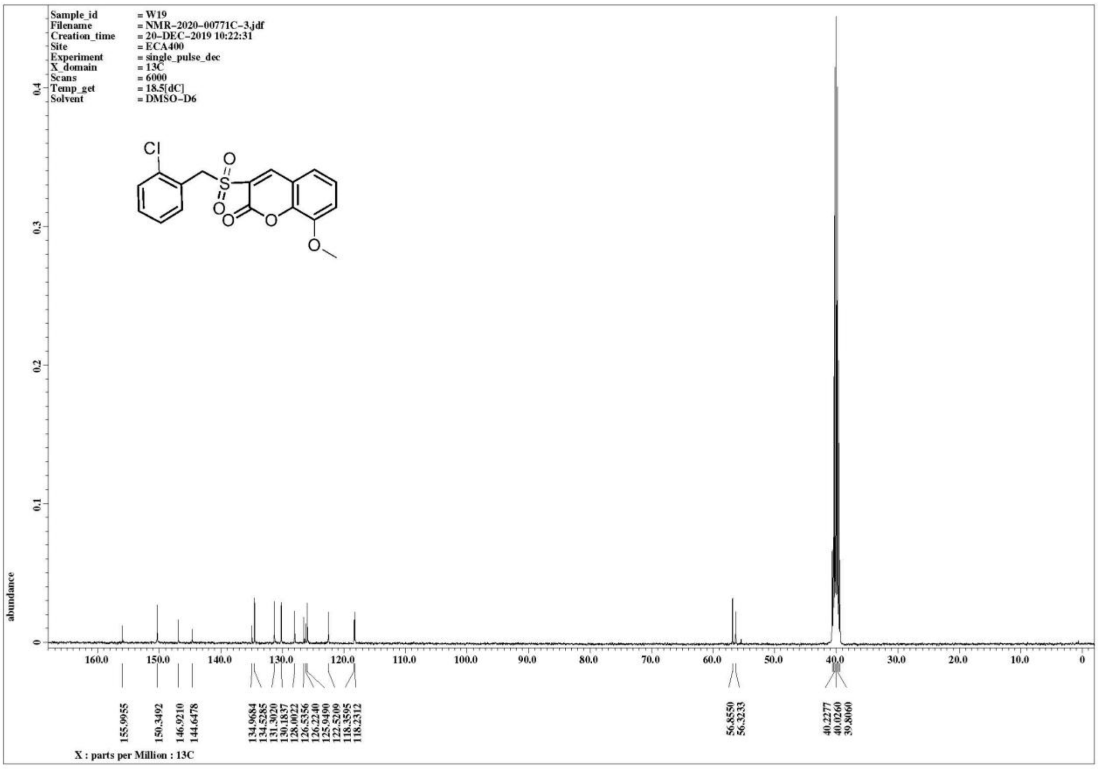

HRMS of Compound **5t**:

Qualitative Analysis Report

**Data Filename**	0596.d	**Sample Name**	FS11
**Instrument Name**	TOF G6230A	**Acquired Time**	2019-12-09
**Acq Method**	YCL.M	**Acquired SW**	6200 series TOF/6500 series
**IRM Calibration Status**	**Success**		
**User Chormatograms**			

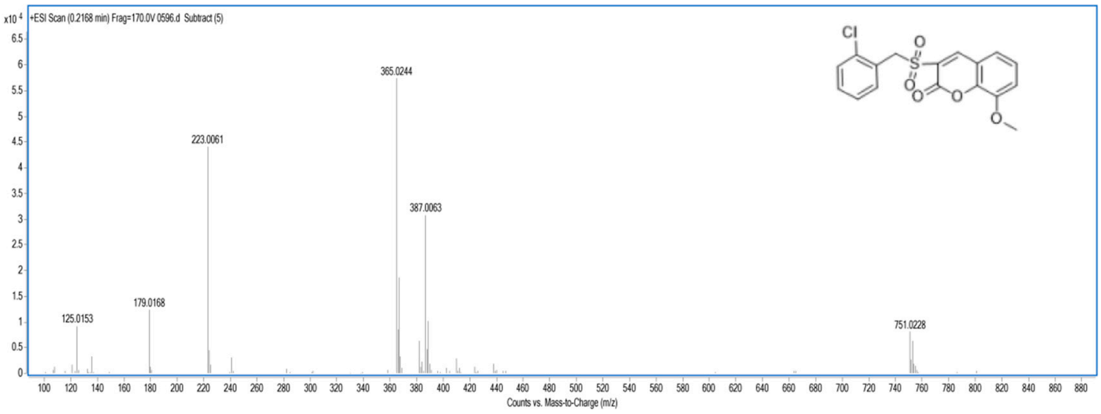

^{1}H-NMR of Compound **5u**:

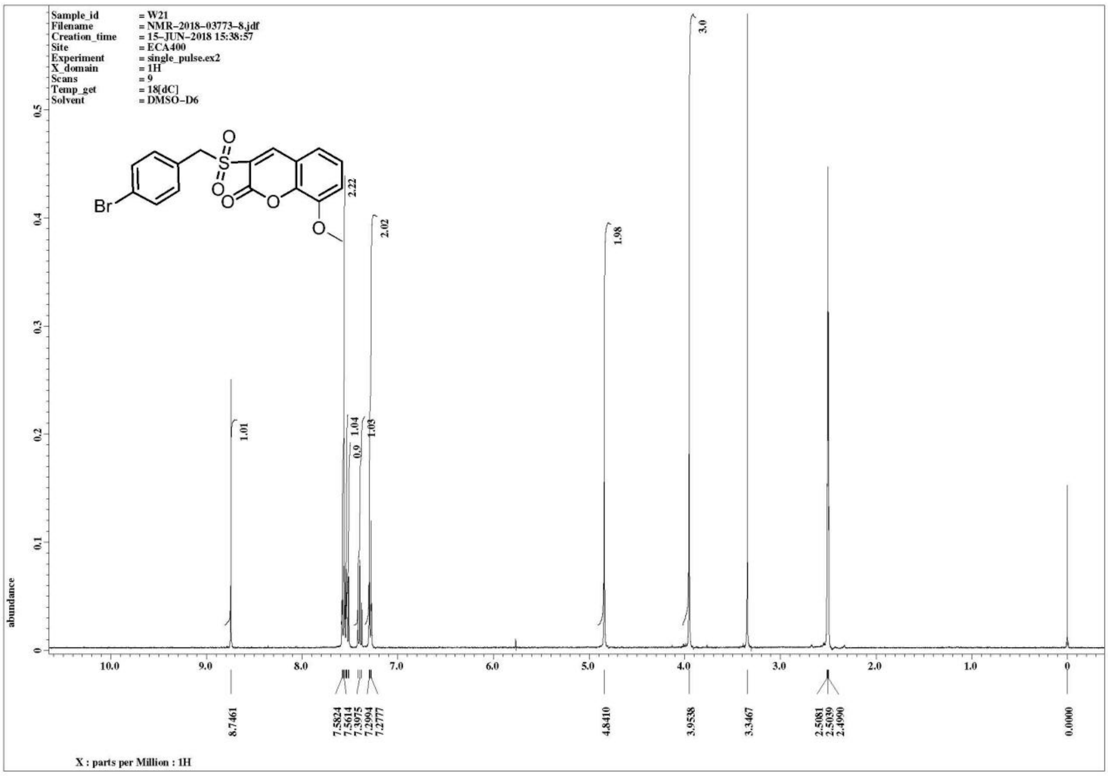

^{13}C-NMR of Compound **5u**:

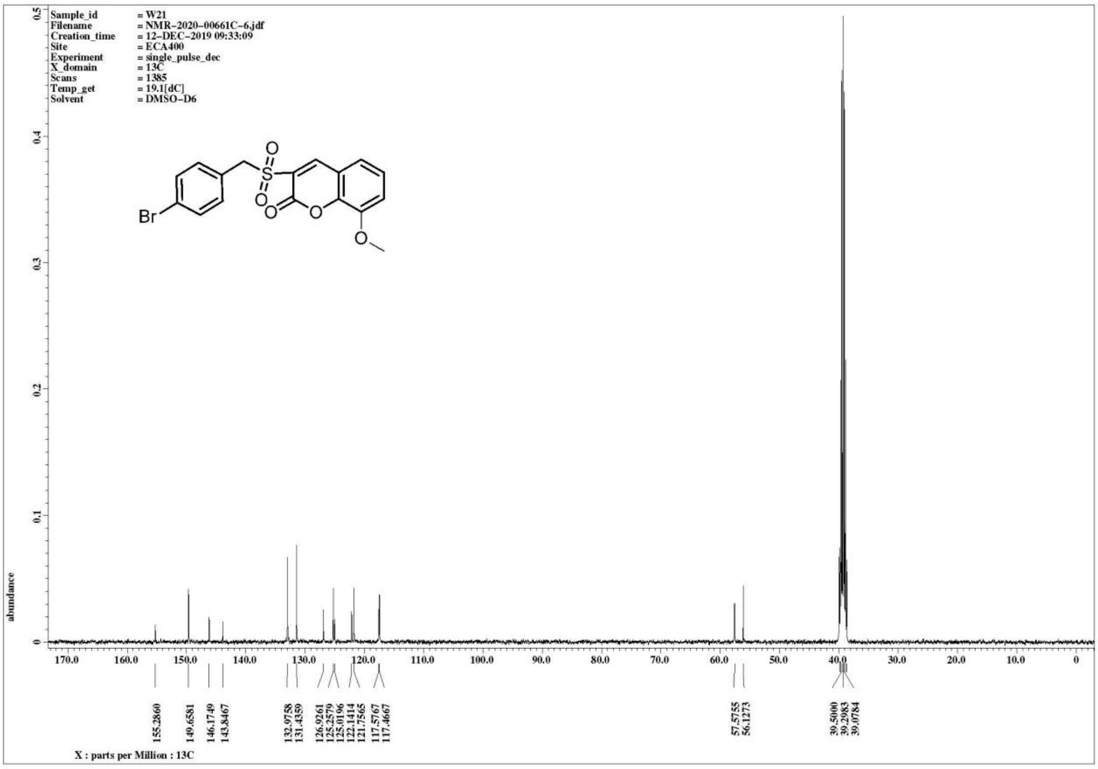

HRMS of Compound **5u**:

Qualitative Analysis Report

**Data Filename**	0597.d	**Sample Name**	FS12
**Instrument Name**	TOF G6230A	**Acquired Time**	2019-12-09
**Acq Method**	YCL.M	**Acquired SW**	6200 series TOF/6500 series
**IRM Calibration Status**	**Success**		
**User Chormatograms**			

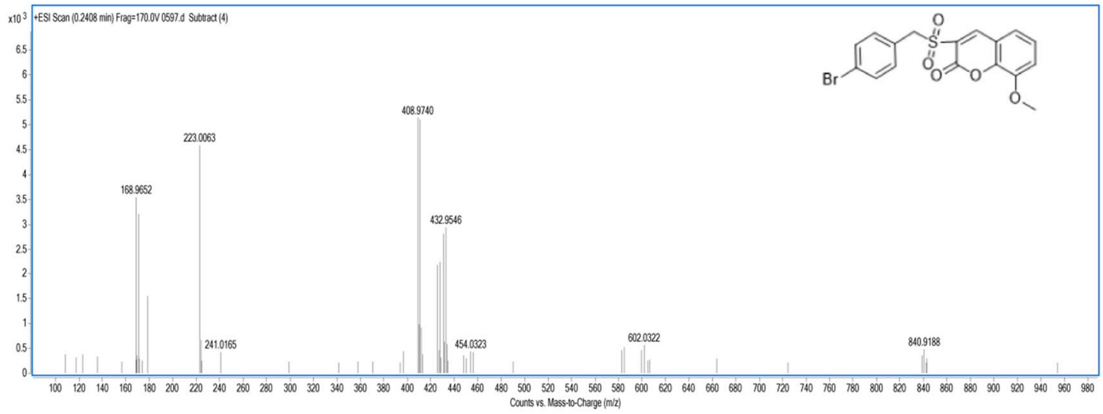

^{1}H-NMR of Compound **5v**:

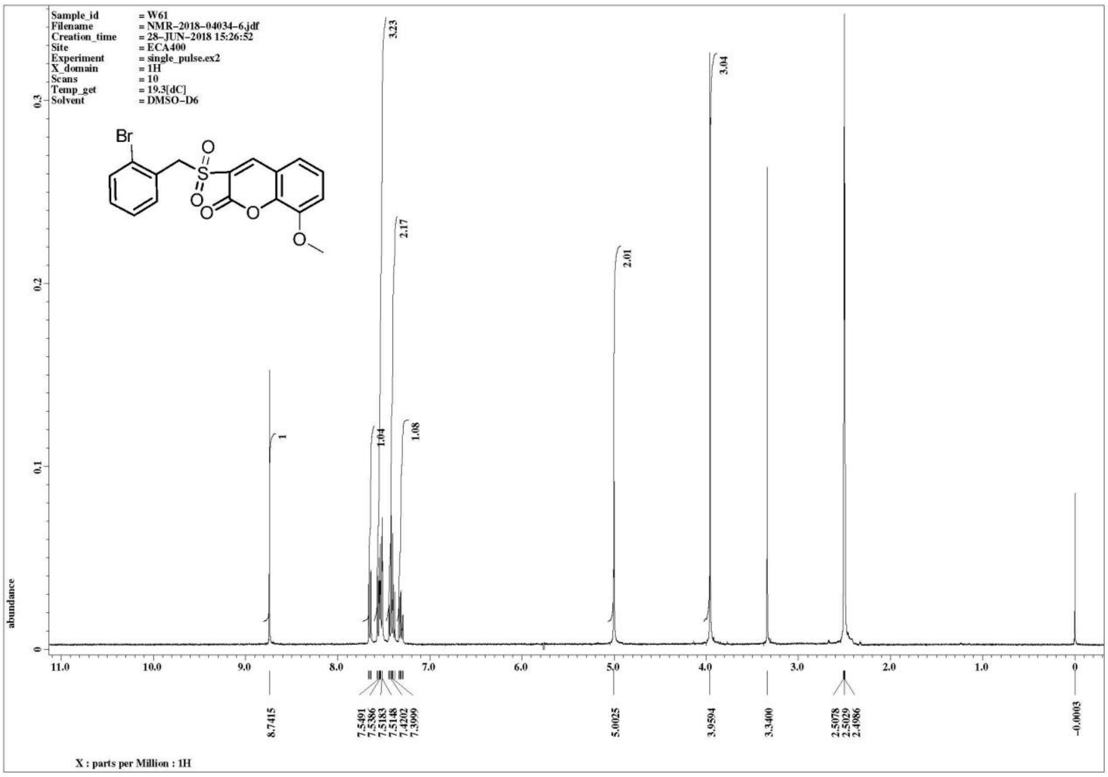

^{13}C-NMR of Compound **5v**:

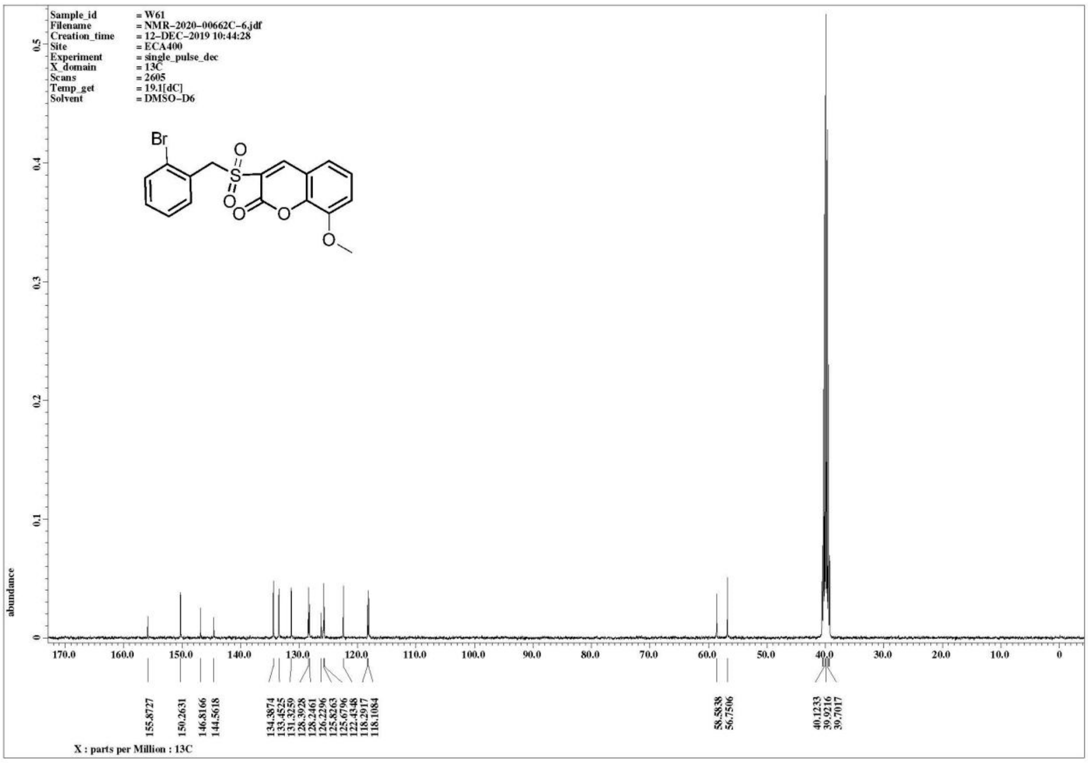

HRMS of Compound **5v**:

Qualitative Analysis Report

**Data Filename**	0598.d	**Sample Name**	FS13
**Instrument Name**	TOF G6230A	**Acquired Time**	2019-12-09
**Acq Method**	YCL.M	**Acquired SW**	6200 series TOF/6500 series
**IRM Calibration Status**	**Success**		
**User Chormatograms**			

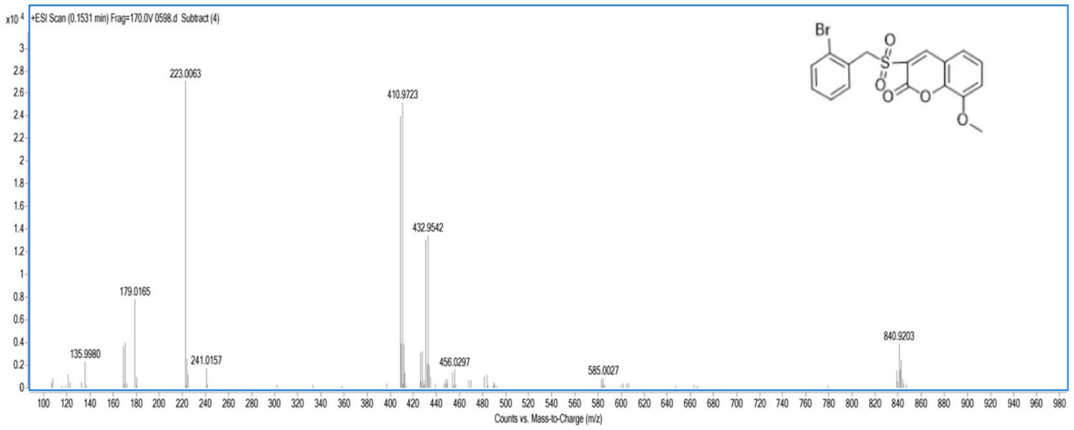

3. Representative fluorescence microscopy images of HUVECs taken 24 hours after exposure to 6.0 Gy irradiation

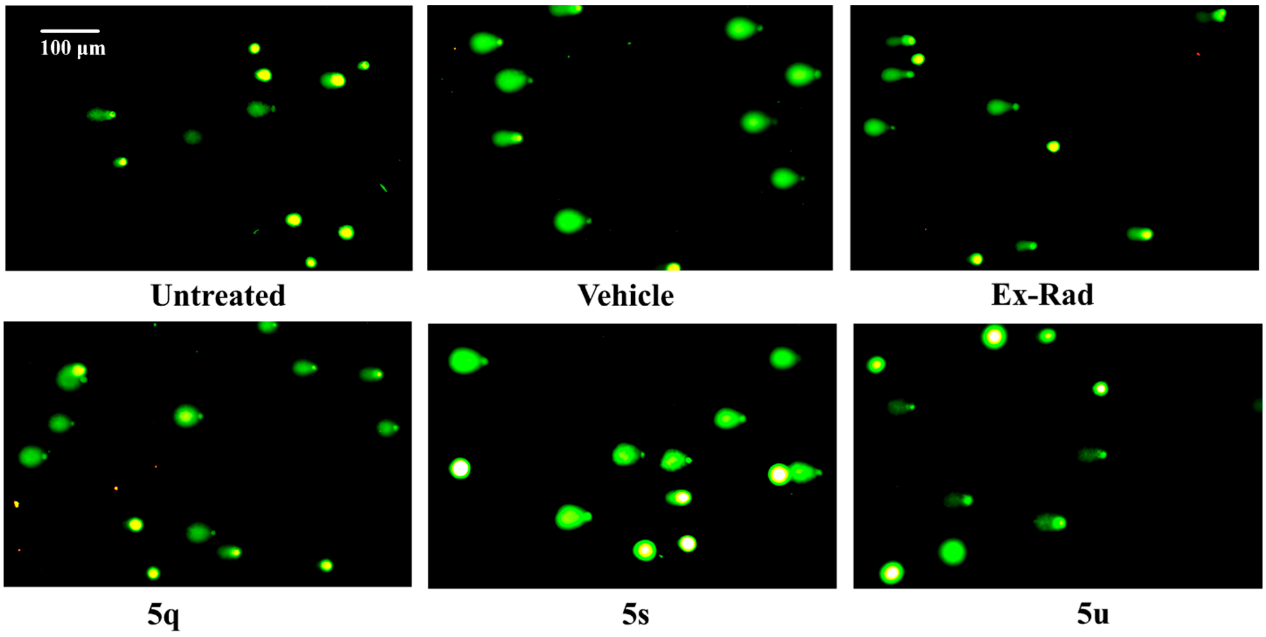

Supplement: Supplementary file 1 [file molecules-31-00487-s001.zip › molecules-4098059-supplementary.pdf]
